# Transcription Start Regions in PTU-intergenic regions drive cell cycle-dependent transcriptional activation events in *Leishmania donovani*

**DOI:** 10.1128/spectrum.01096-26

**Published:** 2026-08-03

**Authors:** Ashish Singh, Vishal Dashora, Arushi Khanna, Swati Saha

**Affiliations:** 1 Department of Microbiology, University of Delhi South Campus 93081, New Delhi, India; CSIR - Institute of Microbial Technology, Chandigarh, Chandigarh, India

**Keywords:** *Leishmania donovani*, trypanosomatid, *Leishmania* transcription, *cis*-acting regulation, *Leishmania* gene regulation

## Abstract

*Leishmania* displays an unconventional mode of transcription, with long clusters of genes being transcribed polycistronically from Transcription Start Regions (TSRs), being processed into monocistronic units prior to translation. It has long been believed that transcription is constitutive: failure to identify consensus sequences across TSRs (except a GT-rich motif supporting transcription in *Trypanosoma brucei*) and absence of canonical eukaryotic transcription factors led to the conclusion that regulation is primarily post-transcriptional, with epigenetics playing a role in triggering transcription initiation. This study stems from our previous findings identifying a few genes to be activated in a cell cycle-dependent manner. Using nuclear run-on assays to analyze nascent transcripts of two chromosomes, chromosomes 2 and 14, we find that while most genes are constitutively transcribed, a subset of genes gets activated at specific cell cycle stages. Reporter assays reveal that this transcriptional activation is driven by the regions immediately upstream of the genes. Sequence analyses of these TSRs lying in polycistronic intergenic regions (PIRs) uncovered a 10-mer GT-rich motif, in synchrony with earlier findings in *T. brucei* identifying a GT-rich motif at bidirectional TSRs. We also identify a second 25-mer motif at these TSRs, and deletion analyses find this motif to be critical for regulating gene expression. The findings of this study reveal that transcriptional events in these unicellular parasites are more complex than believed thus far: not all transcriptional events are constitutive, polycistronic transcription is not the only mode of transcription, and *cis*-acting sequence elements regulate at least some transcriptional events in these parasites.

**IMPORTANCE:**

Endemic to 90 countries, *Leishmania* parasites cause a spectrum of diseases called Leishmaniases. No vaccines for human use are available to date, and the drugs currently used to treat the disease are expensive, have toxic side effects, and have complex administration regimens, with emerging drug resistance compounding problems. Researchers continue to investigate *Leishmania* cellular processes, with the hope of uncovering new therapeutic target sites. Gene regulation in these parasites is unusual, being modulated by various mechanisms, including epigenetic modifications, gene dosage, and post-transcriptional processing. Transcription is typically polycistronic and constitutive, initiating from Transcription Start Regions (TSRs) lying upstream of the first gene in the polycistronic transcription unit (PTU). The work presented here reveals that a subset of genes is transcribed monocistronically in a cell cycle-dependent manner from Transcription Start Regions lying in the PTU-intergenic regions (PIRs), underscoring the complexities of gene regulation in these parasites.

## INTRODUCTION

The group of diseases called Leishmaniases occurs in three forms: cutaneous, mucocutaneous, and visceral. While 90% of the global burden of visceral leishmaniasis (VL) is borne by Brazil, East Africa, and the Indian subcontinent, about 70%–75% of the burden of cutaneous leishmaniasis (CL) is borne by Central Asia, North and East Africa, and Brazil. Endemic to 90 countries, over a billion people live in these areas and are thus at risk of infection, though most people who get infected don’t develop the disease. More than a million new CL cases and ~30,000 new VL cases are reported each year (https://www.who.int/health-topics/leishmaniasis). The different forms of the disease are caused by different species of *Leishmania*, with VL being caused primarily by *Leishmania donovani* in the Indian subcontinent. VL is fatal if not treated in a timely manner, and although there are drugs to treat the disease, emerging threats from rising drug resistance, post-kala-azar dermal leishmaniasis, and *Leishmania*-HIV co-infections continue to give impetus to studies examining the parasite’s physiological processes. Thus, the regulation of the expression of its genes remains an important area of investigation.

**Fig 1:**
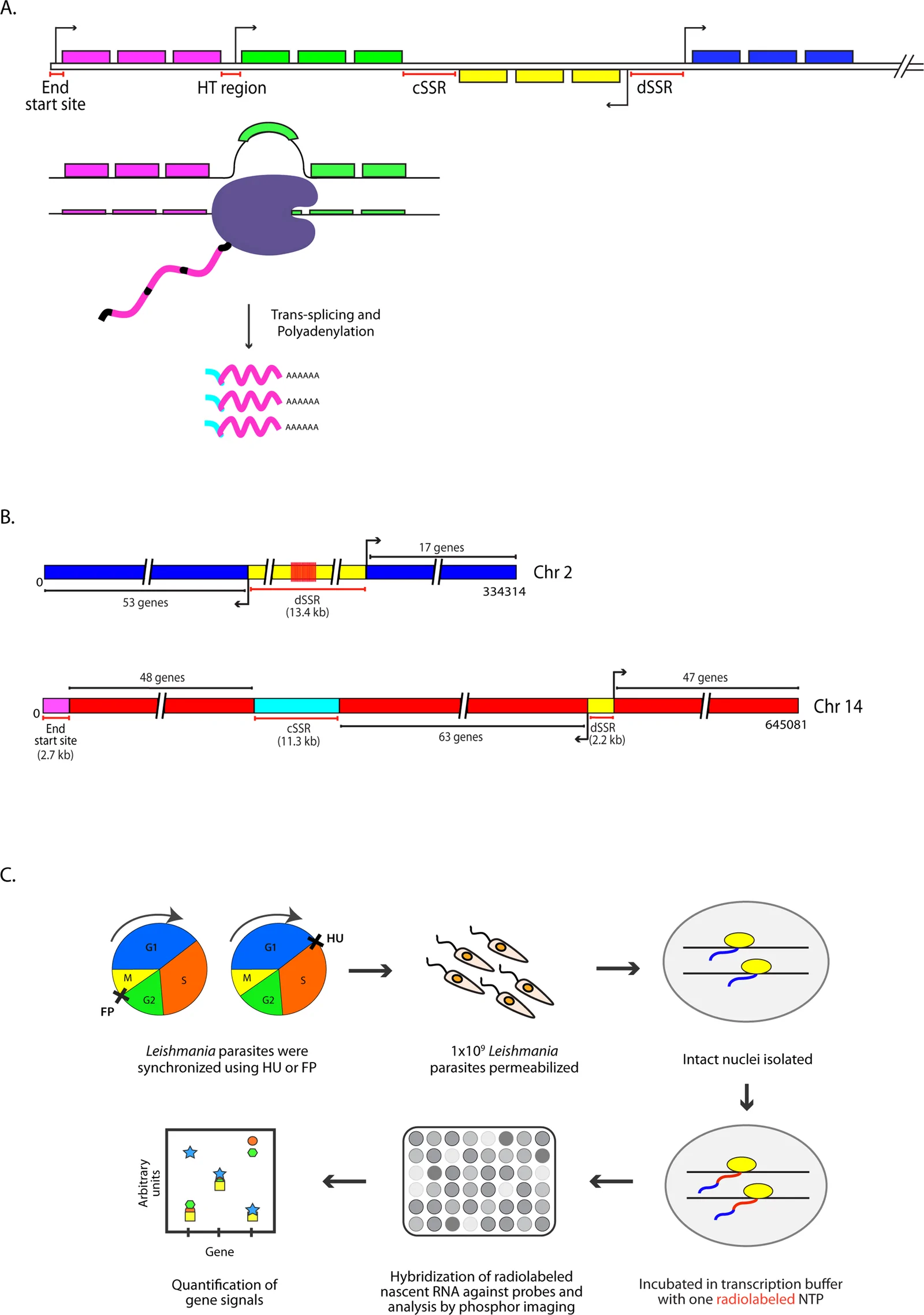
Organization and transcription of the *Leishmania* genome. (**A**) Upper panel: the *Leishmania* genome is organized into long unidirectional arrays of genes, represented by the filled boxes. dSSR: divergent strand switch region where transcription initiates bidirectionally. HT: head-tail sites where transcription of a cluster initiates immediately adjacent to the transcription termination site of the previous cluster on the same strand. End start site: Transcription Start Region at one end of the chromosome. cSSR: convergent strand switch region where transcription of opposing arrays terminates. Lower panel: polycistronic transcription accompanied by post-transcriptional processing: trans-splicing of 5′ SL sequence and 3′ polyadenylation of individual coding units. (**B**) Organization of genes on chromosomes 2 and 14 based on the data publicly available for LdBPK282A1 in the Tritryp DB. Total length of chromosomes in bp is indicated by numerics below the ends. Red boxes in the chromosome 2 dSSR represent SL RNA genes that have been identified to lie in the dSSR of chromosome 2 in LdCL-SL (6), a total of 68 SL RNA genes. (**C**) Workflow of nuclear run-on assays carried out with synchronized parasites. HU, hydroxyurea; FP, flavopiridol.

The organization of genes in trypanosomatids (the family to which *Leishmania* belongs) is highly unusual: tens to hundreds of functionally unrelated genes are arrayed unidirectionally in clusters. Early studies in *Leishmania* revealed that the genes of a cluster are coordinately transcribed from a Transcription Start Region (TSR) upstream of the first gene of the cluster (1, 2). TSRs often initiate bidirectional transcription of oppositely oriented gene arrays. Such TSRs are divergent strand switch regions (dSSRs). Some polycistronic transcription units (PTUs) are arranged end-to-end on the same strand, and TSRs lying between two such clusters are head-tail regions (HT sites). A TSR may also lie at one or the other end of a chromosome (Fig. 1A, upper panel). The polycistronic transcripts so produced are processed into monocistronic units before translation occurs. Post-transcriptional processing involves the trans-splicing of a 39 nt capped spliced leader (SL) RNA to the 5′ end of the individual coding units of the polycistronic transcripts, and polyadenylation at the 3′ ends of the same (Fig. 1A, lower panel [3]). GT-rich tracts have been identified in *Trypanosoma brucei* dSSRs (4); however, no other consensus sequences have been identified across TSRs in any trypanosomatid, supporting the concept of “dispersed” promoters in these organisms. Coupling this aspect with the apparent absence of the canonical regulatory transcription factors typically seen in higher eukaryotes, it is generally thought that transcription from these “dispersed” promoters is constitutive. High-resolution Micro-C contact maps uncovering inter-chromosomal interactions have revealed that the *T. brucei* TSRs are organized into transcription factories in the nuclear space (5).

Differences in levels of transcripts of different genes are believed to largely be the result of post-transcriptional processes (RNA processing, mRNA stability/decay). Hi-C data reflecting the 3D organization of chromosomes in the *T. brucei* nucleus suggest that the genome is spatially arranged to promote efficient processing of transcripts of highly expressed genes, which are found proximal to the SL locus (the array of genes encoding the spliced leader sequence [7]). Events linked to transcription initiation in *T. brucei* have been shown to be globally controlled by histone H4K10 and H2AZ acetylation (8), and in *Leishmania,* the TSRs have been found to be marked with H3 acetylation and H4K10 acetylation (9, 10). TSRs in *T. brucei*, as well as in *Leishmania*, are also marked with H3K4 methylation (11, 12). In addition to post-transcriptional control and epigenetic mechanisms, gene dosage (number of copies of a gene in the genome, which is regulated by genome plasticity) also controls transcript levels in *Leishmania* species, which have a plastic genome (a genome that can expand in some parts, a feature that gives the parasite a survival advantage under adverse conditions), and exhibit mosaic aneuploidy (different chromosomes can have different copy numbers: typically disomic, but can be trisomic or even tetrasomic in clonal populations [13]).

A recent study using PRO-seq to examine genome-wide ongoing transcription in asynchronously growing *Leishmania major* promastigotes has affirmed polycistronic transcription to be the primary mode of transcription in logarithmically growing *Leishmania* species (14). Work from our laboratory has previously unveiled a second tier of transcriptional activation (in addition to the primary tier of polycistronic transcription) of a small subset of genes in *Leishmania donovani* (9). These genes were found to be activated in a cell cycle-dependent manner. The present study examines this second tier of transcriptional activation, with the aim of identifying TSRs lying within the PTUs that govern transcriptional activation of the gene lying immediately downstream of them. Using synchronized cells to capture these events in nuclear run-on assays, we identify such TSRs in two chromosomes, and by determining the minimal region essential for driving gene expression, we further examine these TSRs to identify any critical *cis*-acting sequence elements they may harbor. Our findings underscore the complexities of transcription events in these early diverging unicellular eukaryotes.

## MATERIALS AND METHODS

### *Leishmania* cultures and manipulations

*Leishmania donovani* 1S (Ld1S) promastigotes were grown in M199 medium (Lonza, Switzerland, and Sigma Aldrich, USA) supplemented with fetal bovine serum, hemin, and adenine, as described earlier (15). Lysates were isolated from *Leishmania* promastigotes using the M-PER kit (Thermo Fisher, USA). *Leishmania* parasites were synchronized using hydroxyurea (5 mM) as described earlier (16), and with flavopiridol (5 µM) as outlined previously (17). Flow cytometry analyses were performed as detailed (18). *Leishmania* parasites were transfected as earlier (19), and polyclonal transfectants were selected in M199 complete medium carrying G418 (100 µg/mL).

### Preparation of probes

DNA probes encompassing all the genes on chromosomes 2 and 14 were generated by PCR amplification off Ld1S genomic DNA. For this, primers were designed using the published sequences of the *L. donovani* reference strain LdBPK282A1 ([20, 21] www.tritrypdb.org), as the Ld1S genome sequence is still not available (sequences of primers used are available on request). Accordingly, the ~1 kb regions corresponding to the 5′ end of the genes were amplified using Taq polymerase and cloned into the pGEM-T Easy vector (Promega, USA). All clones were confirmed by single-pass sequencing. The clones were linearized, denatured, and immobilized on a nylon membrane (Hybond N+, GE Healthcare, USA) to prepare dot blots for probing nascent RNA. Every dot blot carried a negative control (empty vector) and a loading/hybridization control (tubulin gene).

### Nuclear run-on assays

Nuclear run-on assays were carried out as described (9). For each reaction, run-on transcription was performed using nuclei isolated from 1 × 10^9^ promastigotes. Accordingly, promastigotes were collected by centrifugation at 1,448*g*, washed once with ice-cold 1× PBS, and resuspended in ice-cold hypotonic buffer (2.5 mL; 0.25 M sucrose, 5 mM HEPES [pH 7.5], 1 mM spermidine, 1 mM EDTA, 1 mM EGTA, 1 mM DTT, 0.1 mM PMSF) before adding Nonidet P-40 (0.5%) and Triton X-100 (0.5%). The mix was vortexed for 35–40 s to lyse the cells, and this was followed by the immediate addition of ice-cold wash buffer (5 mL; 40 mM Tris-Cl, pH 7.5, 0.64 M sucrose, 60 mM KCl, 1 mM spermidine, 1 mM EDTA, 1 mM EGTA, 1 mM DTT, and 0.1 mM PMSF), mixing by quick vortex, and centrifugation to collect the nuclei (3,000 *g*, 10 min, 4°C). The nuclei were washed once in the same wash buffer (5 mL), before resuspending them in 1× transcription buffer (100 μL; 100 mM HEPES [pH 7.5], 50 mM NaCl, 50 mM KCl, 4 mM MnCl_2_, 2 mM MgCl_2_, 0.15 mM spermine, 0.5 mM spermidine, 2 mM ATP, 2 mM GTP, 2 mM CTP, 100 μCi UTP [3,000 Ci/mmole α-^32^P UTP, 10 mCi/mL, BRIT, India], 2 mM DTT, 40 U RNasin [Promega], 25% glycerol). The run-on reaction was allowed to proceed at 26°C for 8 min. A total of 50 U of DNase I (RNase-free, Thermo Fisher, USA) were added to the mix, which was then incubated at 37°C for 5 min, before quenching the reaction by adding Stop buffer (100 μL; 10 mM Tris-Cl [pH 7.5], 10 mM EDTA, 1% SDS, 200 μg/mL proteinase K) (Amresco, USA) and incubating at 37°C for 5 min. After phenol:chloroform extractions, the labeled nascent RNA was purified using G25 microspin columns (GE Healthcare, USA).

For analyzing the nascent RNA by hybridization against probes corresponding to genes of chromosomes 2 and 14, the dot blots were first pre-hybridized (in 1× Denhardt’s solution, 5× SSC, 0.2% SDS, 50% formamide, 100 μg/mL salmon sperm DNA [Sigma Aldrich, USA]) at 42°C for 6 h, before hybridization with the labeled nascent RNA at 42°C for 72 h. In each experiment, for each cell cycle stage, a total of three dot blots were probed (one for the chromosome 2 genes and two for the chromosome 14 genes), with nascent radiolabeled RNA isolated from three reactions carried out simultaneously, pooled, and then split equally among the three blots. The blots were washed (with 2× SSC/0.1% SDS; 20 min/room temperature, and then 20 min/42°C) and exposed for phosphorimaging. The hybridization signals were analyzed using the Amersham Typhoon biomolecular imager (GE Healthcare) and quantified. For each spot representing a single gene from chromosome 2/chromosome 14, the value obtained for the negative control (empty vector) on the same blot was deducted to yield the value for that gene. The same was also deducted from the value of the tubulin gene (loading/hybridization control) on the same blot, to yield the value for tubulin. The gene-to-tubulin ratio was calculated for every gene on a blot. The run-on experiments were done at four stages of the cell cycle. Each experiment was done twice, and the plotted gene: tubulin ratios are the average of the two experiments. The genes are being referred to by their designated numbers in the *L. donovani* reference strain LdBPK282A1.

### Plasmid reporter assays

The plasmid pLEXSY-eGFP-neo3 (Jena Biosciences, Germany) was used as the backbone for reporter assays. The ~1.5 kb upstream regions (URs) of the candidate genes being tested for promoter activity were amplified from Ld1S genomic DNA using primers designed against the LdBPK282A1 genome sequence (sequences of primers available upon request), and the amplicons were cloned into the EcoRI-BglII sites of the vector such that they replaced the promoter of the eGFP gene in the vector. All reporter plasmids so generated were verified by sequencing. The reporter plasmids were transfected into logarithmically growing promastigotes, and polyclonal populations of transfectant parasites were selected using G418 (100 µg/mL). Selection pressure was maintained for 15 days before analysis of reporter gene expression. Analysis of eGFP expression by western blotting was carried out using anti-eGFP antibody (Cat. No. CAB4211, Invitrogen, USA), and of the *neo^r^* gene was performed using the anti-neomycin phosphotransferase II antibody (Cat. No. 4B4D1, Invitrogen, USA). Western blot analyses were performed thrice, and quantitation was carried out using ImageJ software. Graphs represent average values of three experiments. Error bars indicate standard deviation. Statistical significance was determined using Student’s two-tailed *t*-test.

### Direct fluorescence microscopy

Polyclonal transfectant *Leishmania* cultures were maintained under selection pressure for 15 days, and then analyzed for reporter gene expression by direct fluorescence. For this, the logarithmically growing promastigotes were collected by centrifugation at 664*g*, washed with 1× PBS, and fixed with 2% paraformaldehyde at 4°C for 30 min. The fixed cells were collected by centrifugation and resuspended in 1× PBS before spreading ~3–6 × 10^6^ parasites on poly-L-lysine-coated coverslips. The cell spreads were mounted in antifade solution containing DAPI (Vectashield, from Vector Laboratories [USA]). Cells were analyzed for eGFP expression using a Leica TCS SP8 confocal microscope with a 63× (in oil) objective (ex: 488 nm, em: 510 nm). Z-stacks were captured and images analyzed using Leica LAS X software. Microscopic analysis of reporter activity of all constructs was carried out in two independent experiments, and comparable results were obtained.

### MEME analysis

The MEME suite (Multiple Expectation maximization for Motif Elicitation) was used to search for ungapped sequence motifs in the URs (https://meme-suite.org/meme/tools/meme [22]). The search parameters used included: classic discovery mode, DNA sequence alphabet, site distribution anr (any number of repetitions), up to 10 motifs to be identified. Only identified motifs with negative E values were examined, and the search result motifs of least E values were considered. When searching for GT-rich tracts across all 12 TSRs, the width of the motif was set at 10 nucleotides, and the search was carried out by analyzing the [−1 to −1,500] sequences of all 12 TSRs (where −1 was the first nucleotide upstream of the start codon). When searching for motifs specifically in the 4 TSRs selected for further analysis, the width of the motif was set at 10 nucleotides (minimum) and 50 nucleotides (maximum), and the search was carried out by dividing each of the [−1 to −500] sequences into 10 segments of 50 bp each, followed by analyzing the 40 sequences of 50 bp length that were so generated. When searching for the particular presence of Motif II in the URs, the FIMO (Find Individual Motif Occurrences) tool of the MEME suite (https://meme-suite.org/meme/tools/fimo) was used to scan the 24 URs (12 TSRs and 12 non-activated URs), setting the *P* value cut-off at <1 × 10^−6^ (23).

## RESULTS

### *Leishmania donovani* carries cell cycle stage-specific transcription start regions within the polycistronic gene clusters

The ~32.4 Mb *Leishmania donovani* genome comprises 36 chromosomes and is known to exhibit mosaic aneuploidy (24). This genome plasticity is regulated by several factors, such as the medium of growth and conditions, environmental stress, and the extent of passaging of cultures in the laboratory. NGS-based genome sequencing of the Ld1S maintained in our laboratory has revealed that other than chromosome 26, which is trisomic, and chromosome 31, which is tetrasomic, all chromosomes are disomic (25). By maintaining the Ld1S cultures in the same medium and growth conditions, as well as using only early passages (within 6–9 passages), we find this ploidy to be maintained—recent re-sequencing of the laboratory culture done by us has confirmed the same (A. Singh, V. Dashora and S. Saha, unpublished data). In undertaking the search for TSRs lying within the polycistronic gene clusters, and sequences within such TSRs that may govern transcriptional activation, we selected two disomic chromosomes: chr 2 and chr 14, for our study. At the time of initiation of this work, there was no information regarding the Ld1S *de novo* genome sequence, and we used the genome sequence of LdBPK282A1 as our reference ([20]; www.tritrypdb.org). In making our selection of the two chromosomes, we picked a chromosome that was short and had a single dSSR/transcription start region (chromosome 2, which harbors only 70 protein-encoding genes), and a chromosome that had a single dSSR and also had an end TSR (chromosome 14, which carries 158 protein-encoding genes). Chromosome 2 also harbors the cluster of SL RNA genes (Fig. 1B). The Ld1S genome has recently been sequenced using SMRT sequencing (26). While the sequence is not yet available, the authors find that several chromosomes (chromosomes 7, 11, 13, 18, 28, and 29) display genome rearrangements (insertions/deletions, inversions, and translocations) as compared to the *Leishmania donovani* reference strain LdBPK282A1. No rearrangements have been detected in chromosomes 2 and 14.

**Fig 2:**
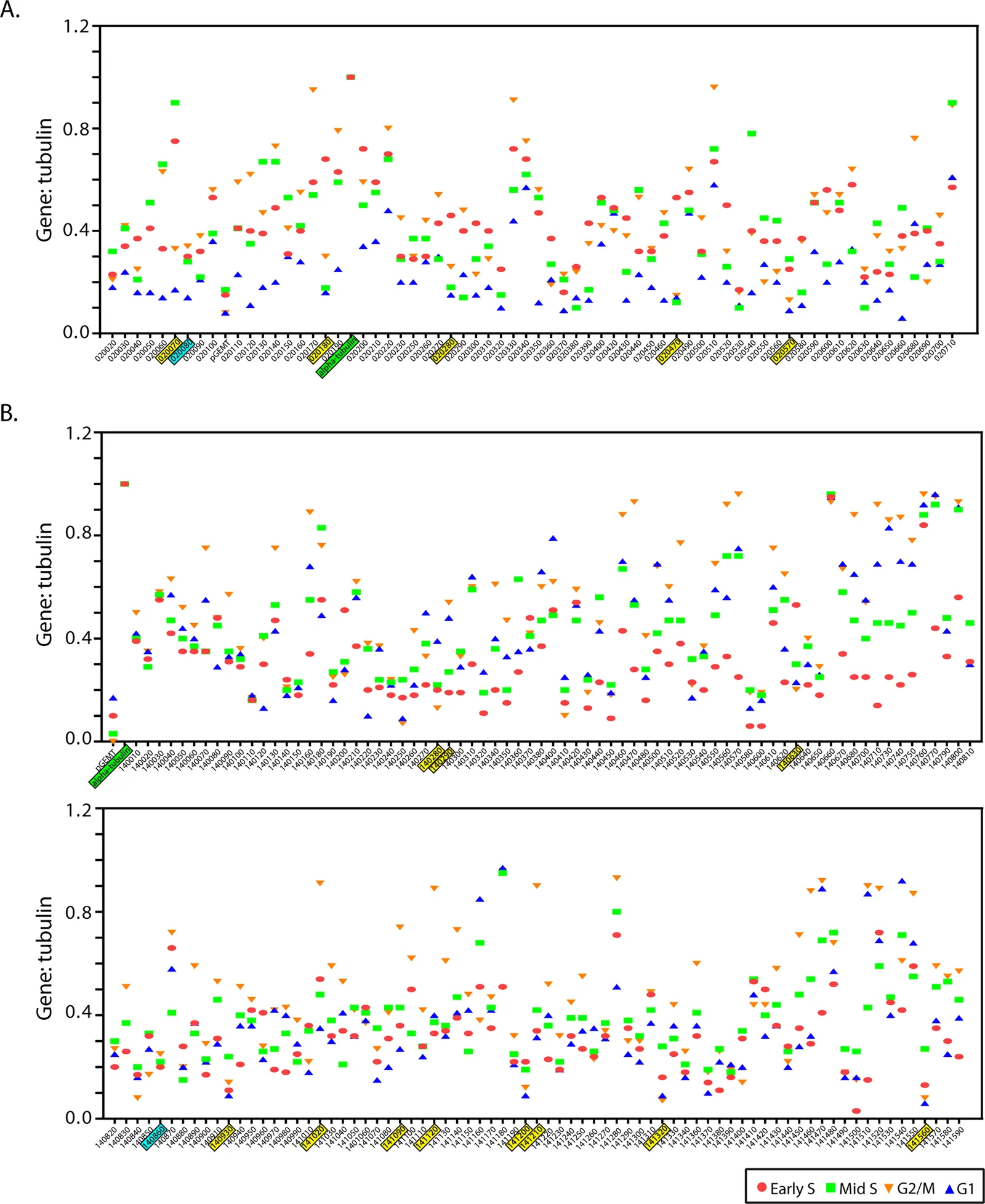
Analyses of nascent transcripts. Analysis of radiolabeled nascent transcripts isolated at each cell cycle stage (after nuclear run-on assays performed with synchronized *Leishmania* parasites), by dot blot hybridizations, followed by phosphorimaging and quantitation. Gene:tubulin ratios of each transcript were determined as detailed in the Materials and Methods. The experiments at each cell cycle stage were performed twice, and average values are plotted here (quantitation raw data in Fig. S7). Key below the figure indicates the symbols used for each of the four cell cycle stages*.* (**A**) Nascent transcripts of chromosome 2 genes. (**B**) Nascent transcripts of chromosome 14 genes. Selected activated genes are highlighted in yellow, selected non-activated genes are highlighted in blue, and tubulin is highlighted in green.

**Fig 3:**
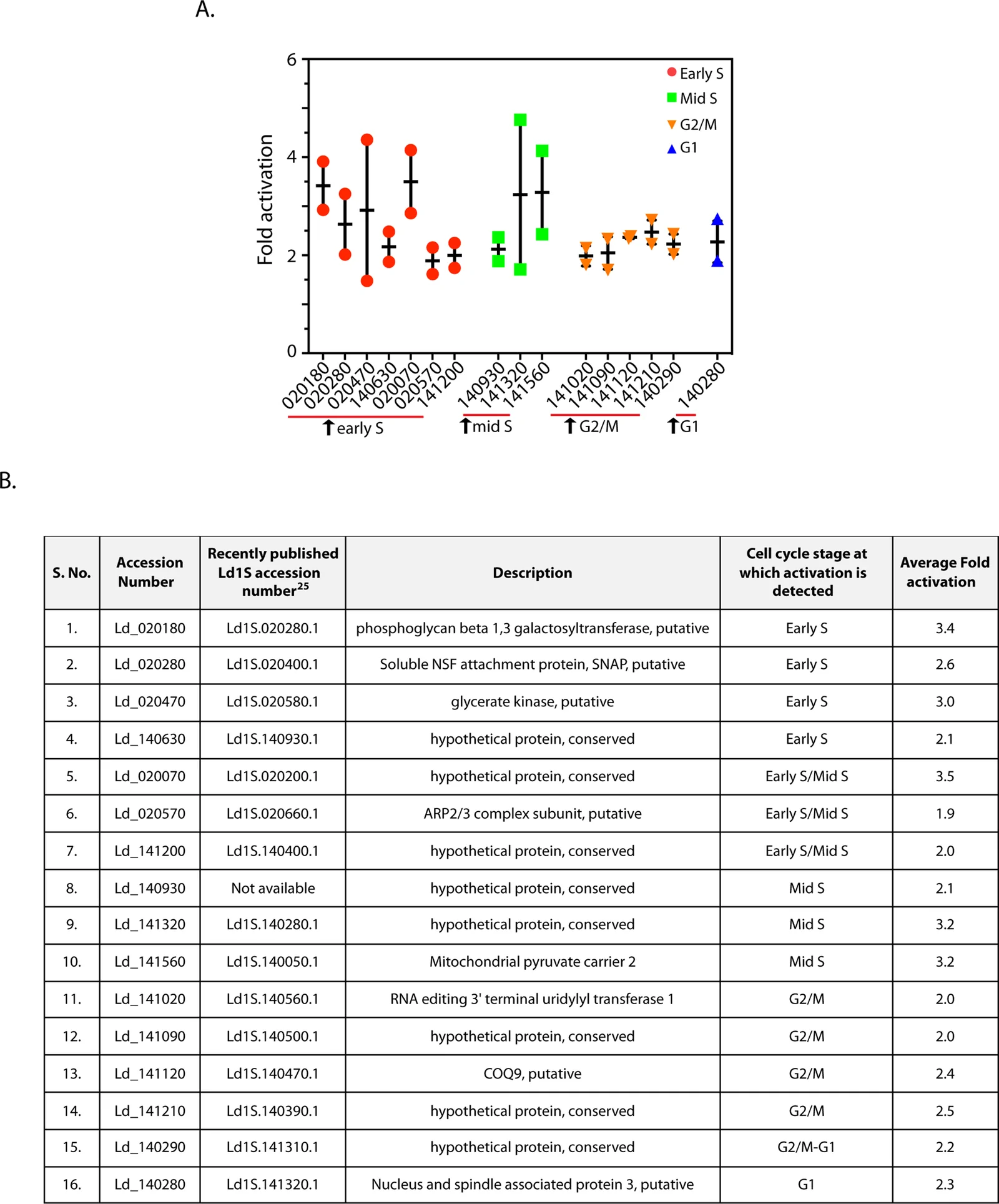
Genes demonstrating an average activation of twofold or more. (**A**) Genes whose average activation at any stage was more than twofold, arranged by cell cycle stage at which activation is seen. Numerics on the x-axis indicate gene accession numbers. The two points for each gene reflect the fold activation for the two individual experiments, respectively, with the horizontal bar representing the average (quantitation raw data in Fig. S8). (**B**) List of genes activated in a cell cycle stage-specific manner. Accession numbers denoted by Ld_ are those of the genes in the LdBPK282A1 reference strain. These numbers have been used throughout the study. The recently published corresponding Ld1S accession numbers are indicated in column 3 of the table. Putative: assumed based on computational analysis.

To identify TSRs lying within the unidirectional gene arrays on both these chromosomes, we adopted the approach of analyzing nascent transcripts using nuclear run-on assays. As our earlier study had found this second tier of transcriptional activation to be cell cycle stage-specific, we carried out the analyses at different cell cycle stages using synchronized cells. Thus, parasites were synchronized at the G1/S boundary using hydroxyurea as earlier (16), released into S phase, and cells were collected 30 min and 3 h after release to isolate nuclei from cells in early S and mid-S phase. Parasites were synchronized at G2/M using flavopiridol as previously (17), released from the flavopiridol-induced block, and collected 30 min and 3.5 h after release to isolate nuclei from cells in G2/M and G1, respectively. Run-on transcription reactions were performed with these nuclei (Materials and Methods; Fig. 1C), and the isolated radiolabeled nascent transcripts were analyzed against probes corresponding to all the genes on both chromosomes, using dot blots (Fig. S1). As is evident, there were considerable variations in signals among genes on the same blot, likely due to variations in the efficiency of hybridization to the different probes, linked to their sequence content and blot washes after hybridization. To normalize for this factor, the signal of every gene was determined relative to tubulin (on the same blot), at every stage of the cell cycle, as described in the Materials and Methods. The relative levels of a particular gene transcript (with respect to tubulin) at all four stages were plotted (Fig. 2A and B) and analyzed for activation at any one stage over and above the other three stages. Fold activation of a gene at a specific cell cycle stage was determined by ascertaining the gene:tubulin ratio at that stage, as a fraction of the mean gene:tubulin ratio at the other three cell cycle stages. Eleven genes on chromosome 14 were found to be activated at a specific cell cycle stage: two in early S (one of which remained activated in mid-S), three in mid-S, five in G2/M (one of which remained activated in G1), and one in G1 (Fig. 3A and B). Five genes on chromosome 2 were similarly activated, all in early S (two of them remaining activated in mid-S as well; Fig. 3A and B). These sixteen genes were scattered around the two chromosomes, suggesting that they were getting activated from the regions lying immediately upstream of them.

### The TSRs within the polycistronic gene clusters display promoter activity independent of their genomic context

**Fig 4:**
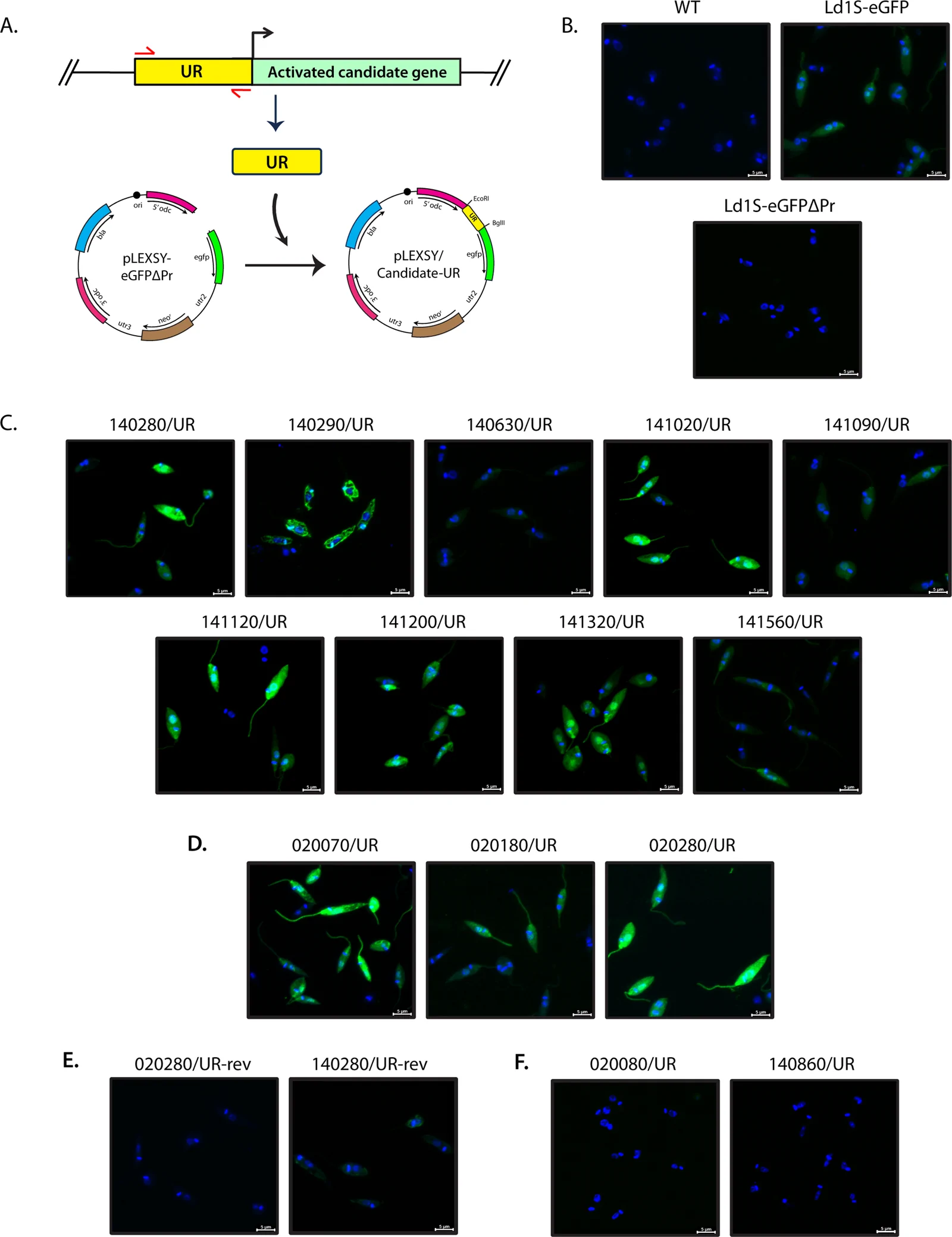
Reporter assays reveal that some PTU intergenic regions (PIRs) display independent promoter activity. (**A**) Regions upstream of genes activated at specific cell cycle stages (upstream regions: URs) were cloned in place of the eGFP native promoter in the pLEXSY-eGFP plasmid. (**B–F**) Analysis of eGFP expression by direct fluorescence microscopy of transfectant *Leishmania* parasites. Parasites were observed using a 63× (in oil) objective. Images of Z-stacks were captured using the Leica TCS SP8 confocal microscope. Images were analyzed using the LAS X software. Magnification bar: 5 µm. Labels above each panel depict the gene number whose UR is being tested for promoter activity. (**B**) WT: untransfected Ld1S parasites, Ld1S-eGFP: Ld1S parasites transfected with the pLEXSY-eGFP plasmid (expressing eGFP under its native promoter), Ld1S-eGFP△Pr: Ld1S parasites transfected with the pLEXSY-eGFP plasmid in which the native eGFP promoter has been deleted. (**C–F**) Ld1S transfectant parasites harboring the reporter plasmid with the test “promoter” upstream of the eGFP gene. (**C**) Reporter plasmids harboring the URs of chromosome 14 candidates. (**D**) Reporter plasmids harboring the URs of chromosome 2 candidates. (**E**) Reporter plasmids harboring URs of candidates in the reverse orientation. (**F**) Reporter plasmids harboring URs of non-activated candidate genes.

The putative (assumed to be, based on the data above) TSRs were examined for their independent ability to drive transcription using plasmid reporter assays. Accordingly, the ~1.0 to 1.5 kb regions lying immediately upstream of the 16 candidate genes were amplified off Ld1S genomic DNA and cloned upstream of the eGFP reporter gene (Materials and Methods; Fig. 4A). The reporter plasmid constructs were transfected into *Leishmania* parasites, and polyclonal transfectant cultures were examined microscopically for reporter gene expression ~15 days after transfection. As seen in Fig. 4B, parasites carrying the original pLEXSY-eGFP plasmid exhibited eGFP expression, which was lost upon deletion of the promoter. eGFP expression was restored upon insertion of the URs of nine of the eleven candidate genes of chromosome 14 (Fig. 4C), and three of the five candidate genes of chromosome 2 (Fig. 4D), although to variable extents. Four of the sixteen candidate URs being tested did not drive eGFP expression, suggesting that either essential features were absent in the regions being tested for promoter activity, or that these URs were only active in their genomic context (Fig. S2). Two candidate URs that drove reporter gene expression were tested in their reverse orientation as well. While one of them did not support eGFP expression, the second supported very weak expression of eGFP (Fig. 4E). This observation was in keeping with earlier findings suggesting low levels of antisense transcription in these parasites (4). The URs of genes that did not exhibit cell cycle stage-dependent activation did not demonstrate promoter activity either (Fig. 4F).

**Fig 5:**
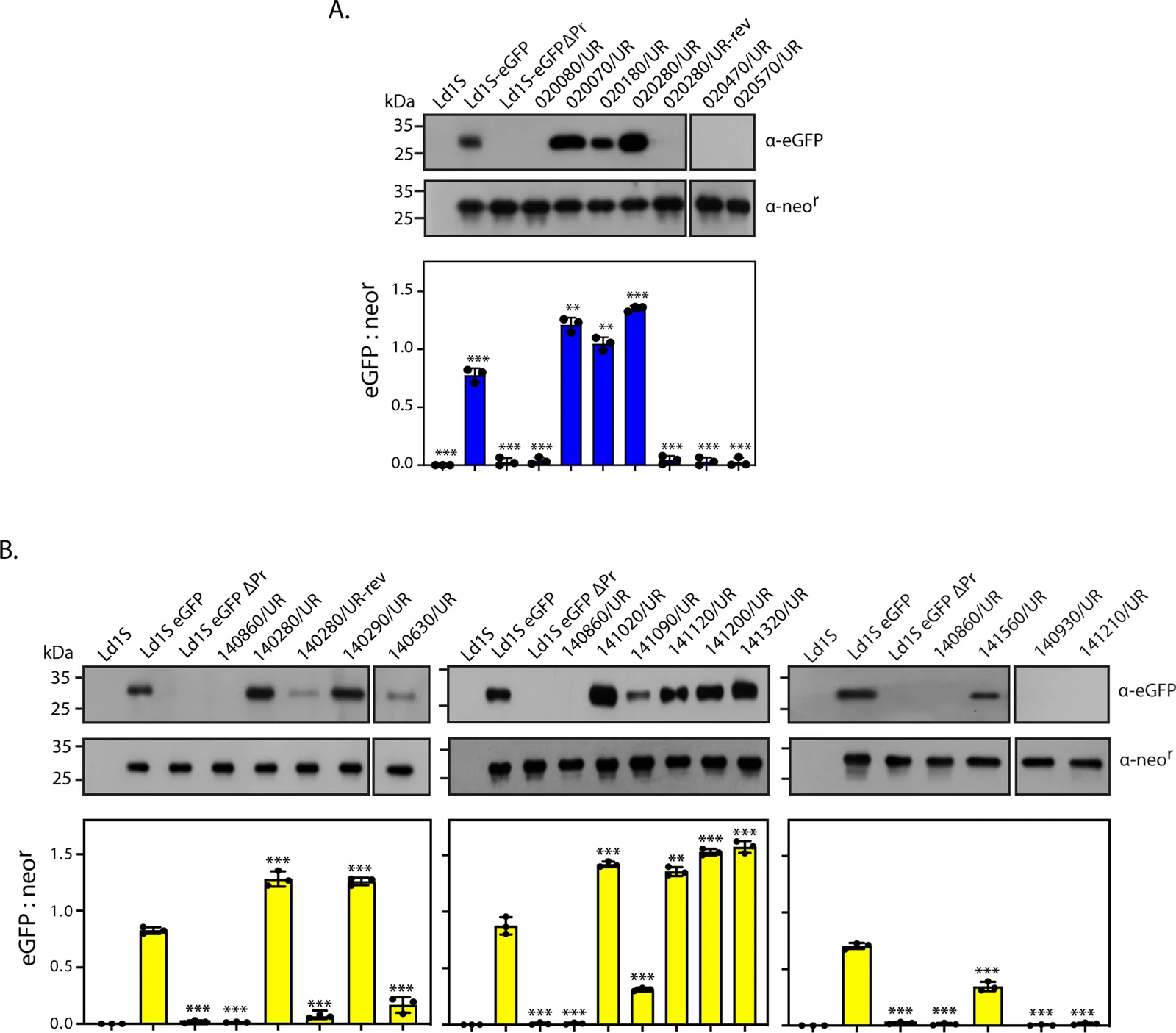
Analysis of whole cell lysates for reporter expression. Western blot analysis of *Leishmania* parasite lysates isolated from 4 × 10^7^ logarithmically growing transfectant cells, using anti-GFP antibodies (1:2,500 dilution). Expression of the *neo^r^* gene (antibody used at 1:2,500 dilution) served as a loading control. (**A**) Analysis of transfectant parasites harboring reporter plasmids testing chromosome 2 URs. (**B**) Analysis of transfectant parasites harboring reporter plasmids testing chromosome 14 URs. Ld1S: untransfected parasites. Ld1S-eGFP: Ld1S parasites transfected with the pLEXSY-eGFP plasmid (expressing eGFP under its native promoter). Ld1S-eGFP△Pr: Ld1S parasites transfected with the pLEXSY-eGFP plasmid in which the native eGFP promoter has been deleted. Numeric labels above blots refer to the gene number whose UR is carried by the reporter plasmid. The experiments were done thrice and blots of one experiment are shown here (raw blots are shown in Fig. S5). Blots of all three experiments were quantitated using ImageJ analysis (https://imagej.net/ij/) and the ratio of eGFP expression relative to expression of the *neo^r^* gene determined (quantitation raw data in Fig. S9). Bar graphs depict the average of three experiments, and error bars indicate standard deviation. Closed circles on bar graphs mark values of individual experiments. Statistical significance with reference to Ld1S-eGFP was determined by the two-tailed unpaired Student’s *t*-test. **: *P* value < 0.005, ***: *P* value < 0.0005.

The varying degrees of eGFP expression seen in the different transfectant populations could be due to two reasons: one, unequal amounts of plasmid DNA being harbored by the cells of the different transfectant populations, leading to gene dosage-related variations in eGFP expression, and two, unequal strengths of promoter activity of the regions being tested. To address this issue, we employed western blot analysis to examine eGFP expression, using the expression of the neomycin resistance gene carried on the reporter plasmid as an internal loading control to account for plasmid copy number differences in transfectant cultures. The experiments were done thrice, the signals quantified using the ImageJ software (27), the eGFP:Neo^r^ ratios determined in each experiment, and average values plotted (Fig. 5A and B). As evident from the data, the extents of eGFP expression in the different populations reflected the differences observed in eGFP fluorescence (Fig. 4), suggesting that these differences were in fact due to the variable strengths of promoter activity demonstrated by the different URs. Collectively, the reporter assays demonstrated that the majority of the URs of the candidate genes had the capacity to act as TSRs independent of their genomic context.

### Critical DNA sequences within these TSRs modulate transcription initiation events

**Fig 6:**
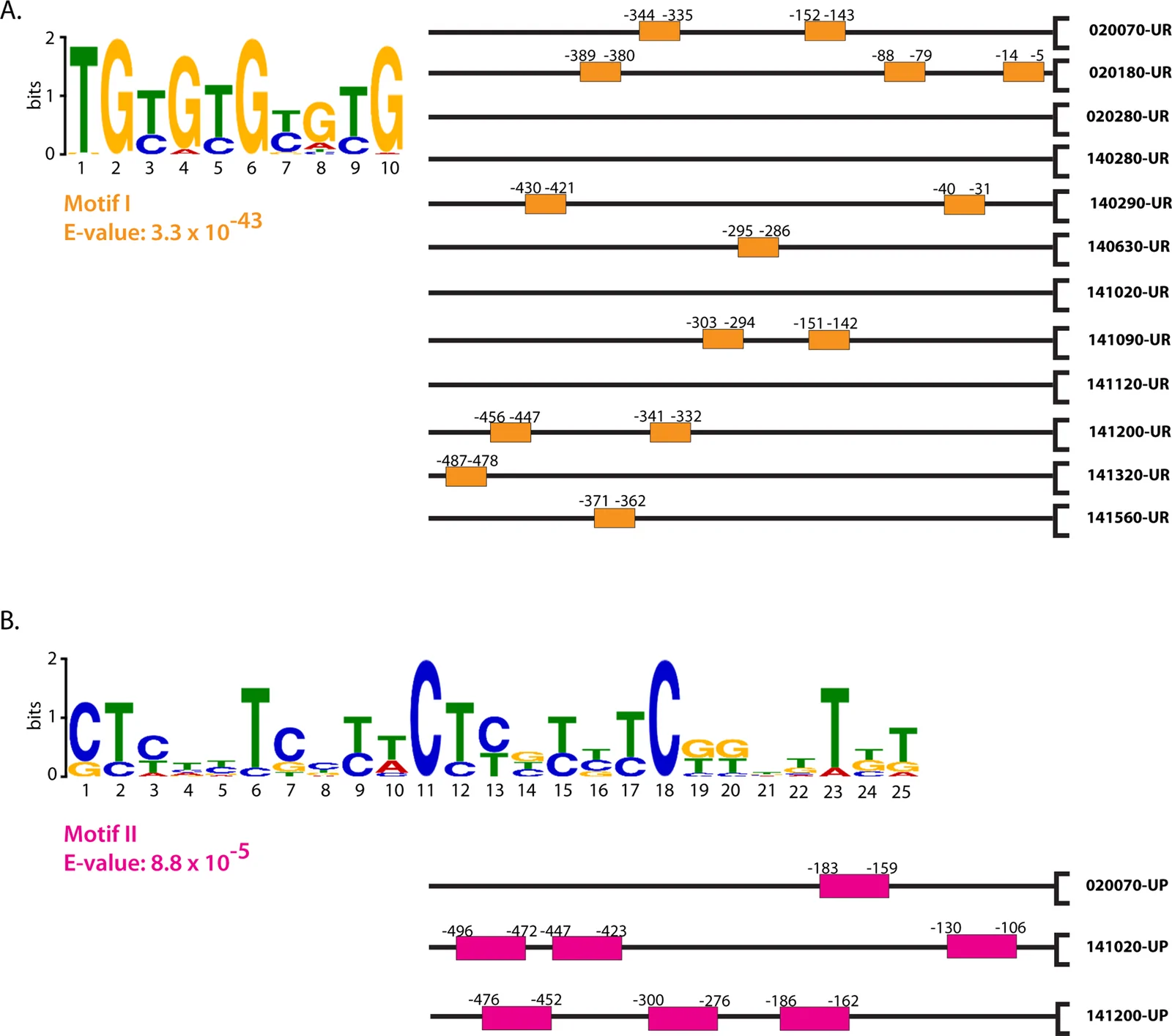
Analysis of sequences of the identified TSRs lying in PIRs. Motifs were searched for and identified using the MEME tool (https://meme-suite.org/meme/tools/meme). −1 refers to the first nucleotide upstream of the genes’ start codons. (**A**) Analysis of sequences of the 12 TSRs. Regions [−1 to −500] are depicted here by a black line, with labels on the side indicating the gene number whose TSR is being analyzed; regions [−500 to −1,500] are depicted in Fig. S3. Motif I: 10-mer GT-rich sequence, indicated by orange boxes on black line. Positions of motifs are indicated by numerics above boxes. (**B**) Analysis of sequences of the candidate TSRs selected for further analysis. TSRs being analyzed are represented by black lines, with labels on the side indicating the gene number whose TSR is being analyzed. Motif II: 25-mer sequence, indicated by magenta boxes on black line. Positions of motifs are indicated by numerics above boxes.

The ~500–1,500 bp regions that lie immediately upstream of the gene start codons (indicated as [–1 to −500/−1,500] in the identified TSRs) were analyzed for sequences that may support transcriptional activation. Wedel et al. (4) identified GT-rich 10-mer tracts in *T. brucei* dSSRs, and by testing synthetic promoters enriched in the 10-mer GT-rich element in reporter assays, the authors found the element supported directional transcription initiation events (4). Upon examining the [–1 to −1,500] regions of the 12 TSRs using the MEME tool (described in the Materials and Methods, [22]), a 10-mer GT-rich element (E value 3.3 × 10^−43^) was identified in all except one of them (henceforth referred to as Motif I; Fig. 6A; Fig. S3, Table S1). Four TSRs were selected for further analysis: the URs of Ld020070, Ld020180, Ld141020, and Ld141200 (hereafter referred to as 020070-UP, 020180-UP, 141020-UP, and 141200-UP). The [–1 to −500] regions of these four TSRs were divided equally into 10 segments of 50 bp each, and these 40 segments were searched for motifs using the MEME tool. A motif of length 25 nucleotides (E value 8.8 × 10^−5^) was identified (henceforth referred to as Motif II; Fig. 6B). A search for Motif II in the [−1 to −500] regions of the remaining 8 TSRs, as well as of 12 URs of genes on chromosomes 2/14 that did not display cell cycle stage activation, using the FIMO tool (Materials and Methods), revealed that while 6 out of the 12 TSRs carried Motif II, only 1 out of 12 URs that did not activate transcription carried the motif (Fig. S4, Table S2).

**Fig 7:**
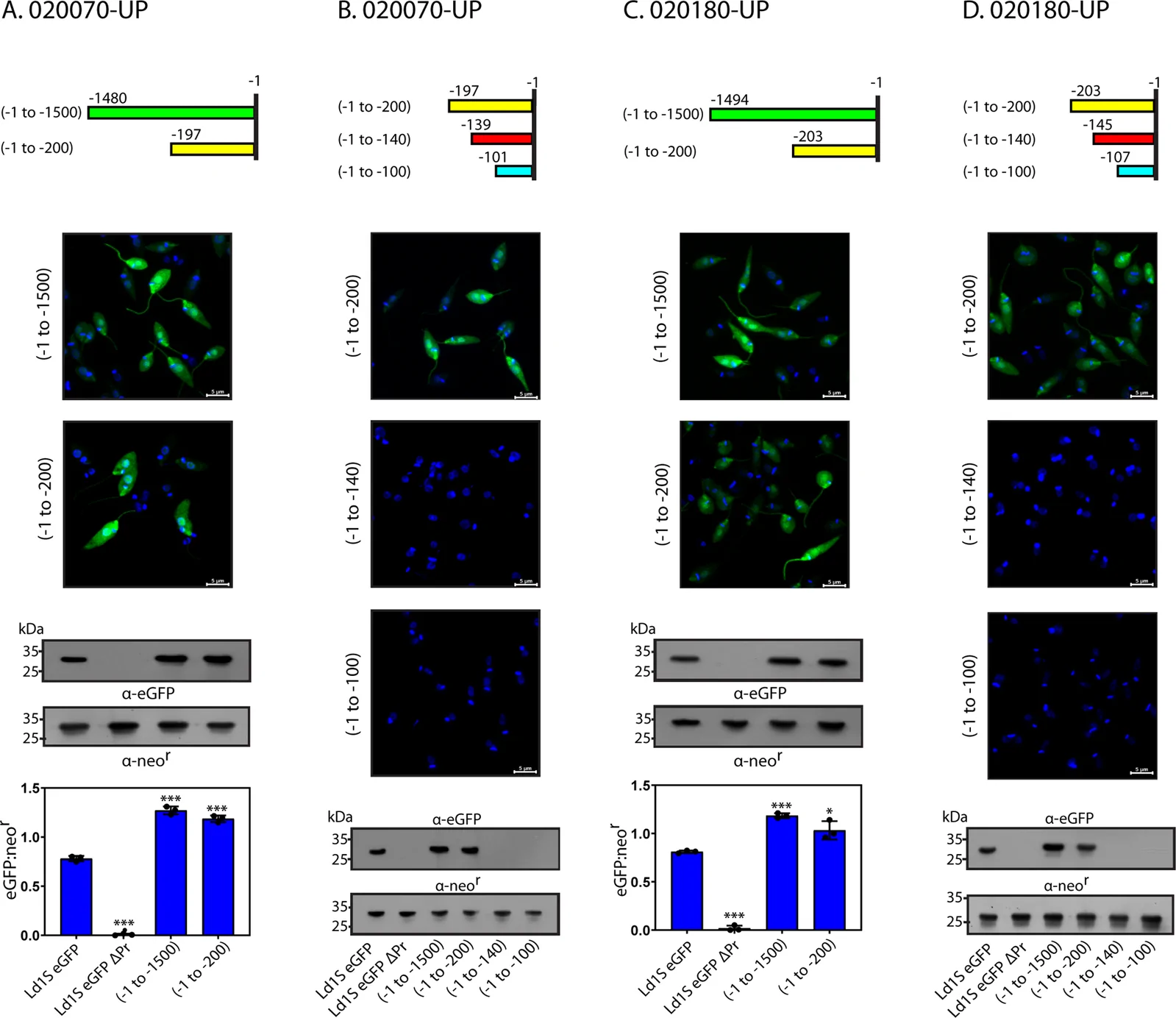
Deletion analysis of two TSRs of chromosome 2. (**A and B**) Analysis of the TSR of 020070. (**C and D**) Analysis of the TSR of 020180. (**A–D**) Upper panels: line diagrams indicating regions being analyzed. −1 refers to the first nucleotide upstream of the genes’ start codons. Middle panels: assessment of reporter expression by microscopic analysis of eGFP fluorescence. Lower panels: assessment of reporter expression by western blot analysis of whole cell extracts isolated from 3 × 10^7^ logarithmically growing transfectant parasites, using anti-GFP (1:2,500 dil) and anti-*neo^r^* (1:2,500 dil) antibodies. Experiments were done three times, and microscopy images and blots of one experiment are shown here (raw blots in Fig. S5). Blots of all three experiments were quantitated using ImageJ analysis (https://imagej.net/ij/) and the ratio of eGFP expression relative to the expression of the *neo^r^* gene was determined (quantitation raw data in Fig. S10). Bar graphs depict the average of three experiments, and error bars indicate standard deviation. Closed circles on bar graphs mark values of individual experiments. Statistical significance with reference to Ld1S-eGFP was determined by the two-tailed unpaired Student’s *t*-test. *: *P* value < 0.05, ***: *P* value < 0.0005.

**Fig 8:**
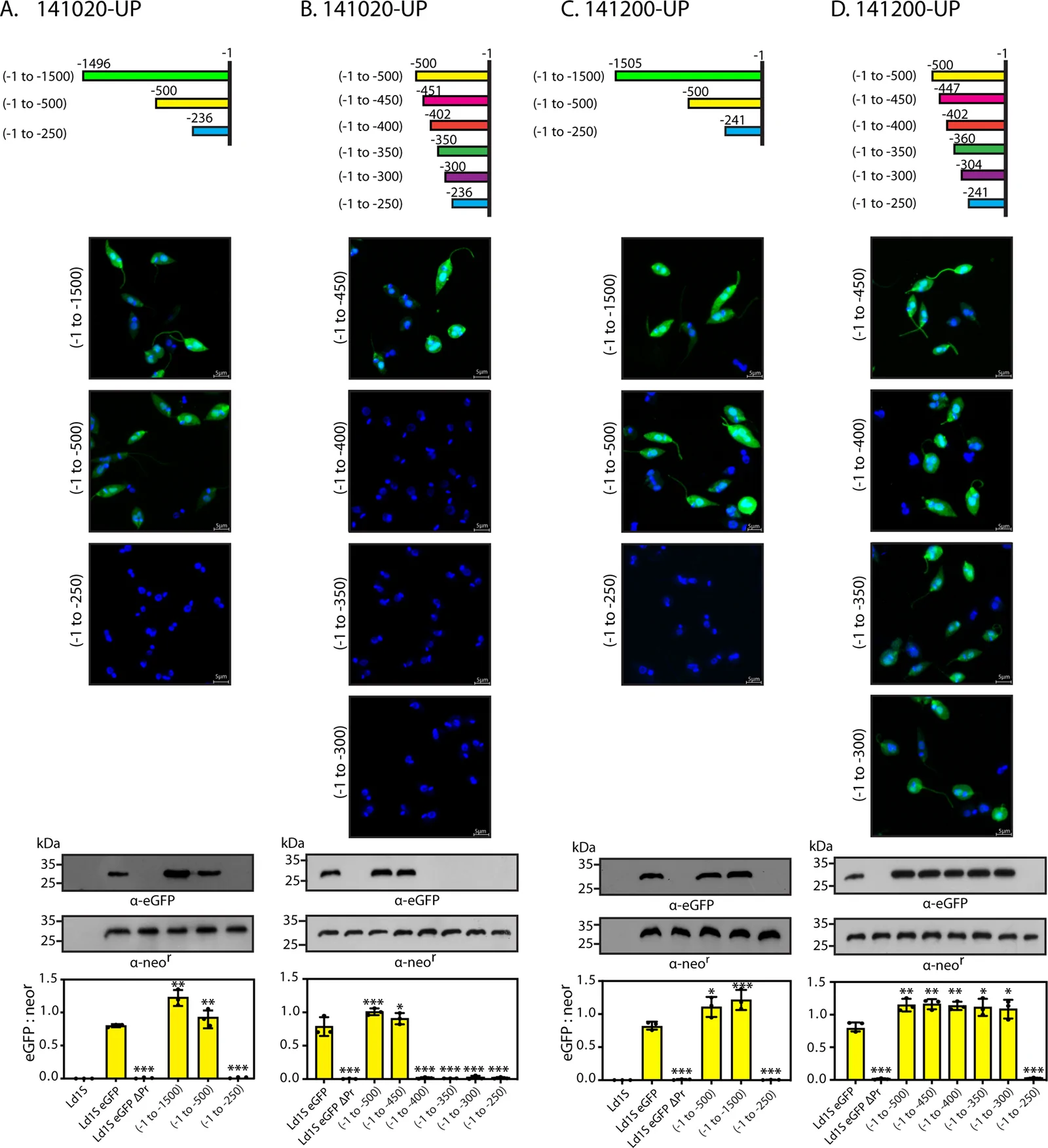
Deletion analysis of two TSRs of chromosome 14. (**A and B**) Analysis of the TSR of 141020. (**C and D**) Analysis of the TSR of 141200. (**A–D**) Upper panels: line diagrams indicating regions being analyzed. −1 refers to the first nucleotide upstream of the genes’ start codons. Middle panels: assessment of reporter expression by microscopic analysis of eGFP fluorescence. Lower panels: assessment of reporter expression by western blot analysis of whole cell extracts isolated from 3 × 10^7^ logarithmically growing transfectant promastigotes using anti-GFP and anti-*neo^r^* antibodies (both at 1:2,500 dil). Experiments were done three times, and microscopy images and blots of one experiment are shown here (raw blots in Fig. S6). Blots of all three experiments were quantitated using ImageJ analysis (https://imagej.net/ij/) and the ratio of eGFP expression relative to expression of the *neo^r^* gene was determined (quantitation raw data in Fig. S11). Bar graphs depict the average of three experiments, and error bars indicate standard deviation. Closed circles on bar graphs mark values of individual experiments. Statistical significance with reference to Ld1S-eGFP was determined by the two-tailed unpaired Student's *t*-test. *: *P* value < 0.05, **: *P* value < 0.005, ***: *P* value < 0.0005.

The four selected TSRs were dissected by creating deletions and linking the deletant TSRs with the reporter gene. Accordingly, the ~200–250 bp regions of the TSRs that lie immediately upstream of the gene start codons were positioned adjacent to the eGFP gene in the reporter construct (Fig. 7A and C and Fig. 8A and C: upper panels). Microscopic analysis revealed that while 020070-UP[−1 to −200] and 020180-UP[−1 to −200] were sufficient to activate eGFP expression (Fig. 7A and C: middle panels), 141020-UP[−1 to −250] and 141200-UP[−1 to −250] did not support gene expression (Fig. 8A and C: middle panels). Therefore, the regions of analyses were extended to ~500 bp upstream of the start codons in the latter two cases, and these were found sufficient for activation of eGFP expression (Fig. 8A and C: middle panels). Western blot analyses confirmed reporter gene expression, and no significant differences in levels of reporter gene expression were apparent in the case of the 020070-UP[−1 to −200] and 020180-UP[−1 to −200] deletants when compared with the respective full-length TSRs (Fig. 7A and C: lower panels). However, while the activity of 141200-UP[−1 to −500] in supporting gene expression was also comparable to that of the full-length TSR, the 141020-UP[−1 to −500] deletant showed a significant decrease in its ability to drive gene expression, implying that sequences lying between −500 and −1,500 must contribute, perhaps serving as enhancers (Fig. 8A and C: lower panels).

Nested deletions were now created between ~100 and 200 bp upstream of the start codons in 020070-UP and 020180-UP (Fig. 7B and D: upper panels). In both cases, it was found that the [−1 to −100] regions did not support reporter expression, nor did the [−1 to −140] regions (Fig. 7B and D: middle and lower panels). The [−140 to −200] regions thus seem to be critical. In creating nested deletions between ~250 and 500 bp upstream of the start codons in 141020-UP and 141200-UP, it was observed that while ~450 bp of upstream region was needed to drive gene expression from 141020-UP, with a further 50 bp deletion leading to complete loss of expression (Fig. 8B: middle and lower panels), the [−1 to −300] region was sufficient to activate expression from 141200-UP, with no expression on deletion of a further 50 bp (Fig. 8D: middle and lower panels). Thus, the [−400 to −450] of 141020-UP and [−250 to −300] of 141200-UP appear to be critical. The levels of expression in 141020-UP[−400 to −450] and 141200-UP[−250 to −300] were comparable to their [−1 to −500] counterparts (Fig. 7B and D and Fig. 8B and D: lower panels showing western blots).

**Fig 9:**
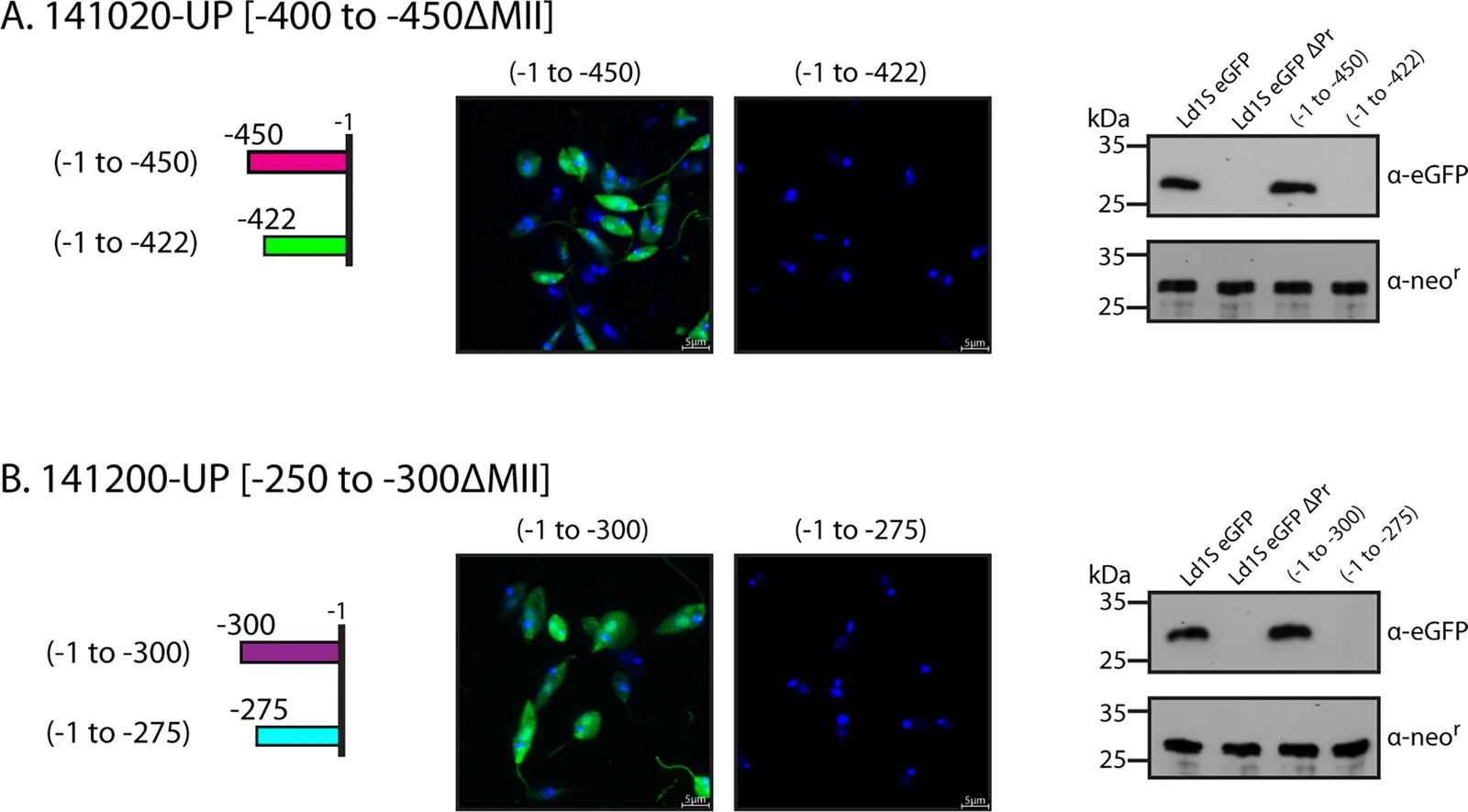
Analysis of the role of Motif II in transcriptional initiation. (**A**) Analysis of 141020-UP[−400 to −450△MII]. [−1 to −422]: 141020-UP[−400 to −450△MII] construct. (**B**) 141200-UP[−250 to −300△MII]. [−1 to −275]: 141200-UP[−250 to −300△MII] construct. Left panels: line diagrams depicting regions being analyzed. −1 refers to the first nucleotide upstream of the genes’ start codons. Middle panels: assessment of reporter expression by microscopic analysis of eGFP fluorescence. Right panels: assessment of reporter expression by western blot analysis of whole cell extracts isolated from 3 × 10^7^ logarithmically growing transfectant promastigotes using anti-GFP and anti-*neo^r^* antibodies (both at 1:2,500 dil) (raw blots in Fig. S6).

In analyzing the segments 020070-UP[−140 to −200], 020180-UP[−140 to −200], 141020-UP[−400 to −450], and 141200-UP[−250 to −300] for the presence of Motifs I and II, it was observed that while Motif II was identifiable in segments 020070-UP[−140 to −200], 141020-UP[−400 to −450], and 141200-UP[−250 to −300], Motif I was present only in 020070-UP[−140 to −200] (Fig. 6B and A). However, Motif I was found elsewhere in the [−1 to −500] regions of 020180-UP and 141200-UP (Fig. 6A), and in the [−500 to −1,500] regions of 141020-UP (Fig. S3). The copies of Motif I identified between −500 and −1,500 of 141020-UP may be the sequences contributing to activation of expression, whose loss in the [−1 to −500] mutant leads to significant downregulation of reporter expression. As Motif I is a GT-rich 10-mer tract whose importance has been previously demonstrated by Wedel et al., we turned our attention to Motif II and assessed its importance in driving gene expression by creating mutants of two TSRs: 141020-UP[−400 to −450△MII] and 141200-UP[−250 to −300△MII]. Upon testing these mutants in reporter assays, we found that loss of Motif II in both cases led to abrogation of reporter expression, signifying the importance of the motif in regulating gene expression (Fig. 9A and B).

Collectively, the data presented above support the theory that TSRs in *Leishmania* not only lie upstream of PCGs but can also lie at PTU intergenic regions (PIRs), activating transcription of the adjacent gene. The activity of these TSRs appears to be controlled at least in part by the sequences lying within them.

## DISCUSSION

Transcription in *Leishmania* species has long been known to be primarily polycistronic, with unidirectionally arrayed clusters of tens to hundreds of genes being coordinately transcribed from Transcription Start Regions that can be several kb in length (1, 2). Recent studies using modern PRO-seq methodology in axenic logarithmically growing cultures of *L. donovani* have revealed that transcription is primarily constitutive in both promastigotes and amastigotes and is life cycle stage-independent, with no differences between individual PTUs of promastigotes versus amastigotes (28). The dynamics of chromatin structure in promastigotes versus amastigotes have been probed using ATAC-seq, and the data obtained reveal that TSRs in fast-growing promastigotes are predominantly euchromatic, but in slower-growing amastigotes are more heterochromatic (29). Two primary features govern transcriptional activation from promoters: one, the local chromatin environment, including nucleosomal composition and histone modifications that make the DNA more accessible, and two, the sequences at the core promoter, which serve as a landing pad on which the transcription machinery is built up through sequence-specific recognition by specific factors. No consensus sequences were identified across trypanosomatid TSRs in early studies, and for a long time, it was believed that the activation of polycistronic transcription at the TSRs was governed only by the chromatin environment and epigenetic modifications. TSRs in *T. brucei* have been found to be enriched in histone variants H2AZ and H2Bv, and these variants are hyperacetylated on their C-terminal tails. Reduction in H2AZ C-terminal hyperacetylation by depletion of the histone acetyltransferase HAT1 extensively reduces global transcript levels (8). *T. brucei* TSRs are also found to be enriched in H4K10 acetylation, and lowering the levels of this acetylation by depletion of histone acetyltransferase HAT2 results in a shift in the sites at which the transcripts start in *T. brucei,* although there is no overall reduction in the amounts of transcripts produced (8). While TSRs are also marked with H3K4 methylation, this modification mark has not yet been directly demonstrated to play a role in modulating transcriptional events in these organisms (11, 12). Our laboratory has previously identified H4K10 acetylation peaks at *L. donovani* dSSRs as well, with depletion of LdHAT2 reducing H4K10 acetylation at the dSSRs but not impacting global transcript levels (9). Our work also uncovered a second tier of transcriptional activation of a small subset of genes, and this tier functioned in a cell cycle stage-specific manner (9).

The present study was undertaken to explore the second tier of transcriptional activation, with the aim of identifying TSRs and critical *cis*-acting elements therein, lying within the polycistronic transcription units (at PTU intergenic regions: PIRs). Rooted in our previous findings that this subset of genes is activated at specific cell cycle stages, we isolated nascent transcripts at the different cell cycle stages and identified the genes which were getting activated at one or other stage (Fig. 2 and 3). Presuming them to be activated from their own promoters on the basis of the fact that they were scattered around the genome, the putative TSRs upstream of these candidate genes were analyzed for promoter activity using reporter assays (Fig. 4 and 5). At least 75% of the analyzed TSRs exhibited independent promoter activity in plasmid reporter assays, and while episomal reporter assays do not capture the impact of chromatin architecture and genomic context that may influence TSR activity, a dissection of four of these TSRs delineated the ~50–60 bp regions critical to activation of gene expression (Fig. 7 and 8). A 25 bp motif was identified within these ~50–60 bp regions in three out of these four cases, and this motif was determined to be essential for driving gene expression (Motif II; Fig. 6B and 9). Additional copies of this motif were also found elsewhere in the [−1 to −500] regions of two of these TSRs, pointing toward the underlying complexities of regulation through this motif. As the present study has not investigated what proteins, if any, bind to this motif, its mechanistic role in transcriptional activation remains uncovered. While the motif may serve as a platform for recruiting essential components of the transcriptional machinery, including hitherto unknown protein players, it is also possible that this sequence somehow serves as a marker for one or more epigenetic modifications that may modulate transcription. The impact of this sequence on nucleosomal positioning also needs to be examined—a more open chromatin environment supported by this sequence could promote transcriptional activation. A 10-mer motif that was identified in the [−1 to −500] regions of several of the 12 TSRs was found in only one of the critical regions of the four tested TSRs (Fig. 6A), although it was found at other regions of these TSRs (Table S1), suggesting that rather than being an essential motif *per se,* it might play a conformational role. Cordon-Obras et al. have found that conserved poly-T and poly-GTGT elements at the PolII peak of one of the *T. brucei* dSSRs lie beyond the essential core promoter region (30).

The results of our study, along with data from two other recent studies in *T. brucei*, lead toward a paradigm shift in our thinking regarding the way trypanosomatid transcriptional events are regulated. Our study reveals that while the vast majority of genes appear to be constitutively transcribed in *Leishmania* species, several genes are transcriptionally activated in a cell cycle stage–specific manner. Of the 16 genes identified in this study, 8 are hypothetical proteins sharing no similarity with other eukaryotic proteins. Of the remaining eight, several are critical for parasite function. Phosphoglycan beta 1,3-galactosyltransferase plays a crucial role in glycosylating the *Leishmania* surface glycolipid lipophosphoglycan, and its deficiency impairs the parasite’s interactions with the insect midgut, a vital link in the transmission chain (31). Soluble NSF attachment proteins regulate the secretion of exosomes and are also believed to play a role in the parasite attachment to the insect midgut (32). The ARP2/3 complex is believed to play a role in nucleating the branched actin networks driving endocytosis (33), a process critical to survival in the insect host, as *Leishmania* do not synthesize heme and must acquire it through the hemoglobin it endocytoses from the insect blood meal. These genes are additionally activated in S phase, possibly preparing for increased levels of these proteins as the parasite nears the completion of binary fission. The RNA-editing 3′ terminal uridylyl transferase 1 in *Leishmania* is an essential enzyme catalyzing the addition of uridine residues to the 3′ end of guide RNAs, a critical feature of insertion/deletion of uridines during mRNA editing (34). While no information is available regarding the timing of RNA editing, the additional activation of the gene encoding the enzyme in G2/M suggests the possibility of a significant number of RNA editing events occurring in this stage. This study was carried out with logarithmically growing promastigotes in the non-infectious procyclic stage. We were unable to evaluate ongoing transcriptional events in stationary-phase promastigotes, which are in the infectious metacyclic stage, as metacyclic promastigotes do not synchronize reproducibly—metacyclics die in large numbers by the time the incubation period in the drug is complete, and do not always get released from the drug-induced arrest.

This study also suggests that *Leishmania*, like other trypanosomatids, transcribes its genome primarily polycistronically, but this is not the only mode of transcription: some genes within the polycistronic clusters are transcribed from their own promoters as well. As the data presented here are limited by the fact that this was not a genome-wide analysis and restricted to two chromosomes only, in-depth studies will have to be carried out to assess the extent of PIR-driven transcription initiation events across the genome. While dSSR/HT-associated TSRs are enriched with the histone variants H2AZ and H2Bv (which are hyperacetylated) and histone modification marks, such as H4K10Ac and H3K4me3, it remains to be seen if these histone variants and modifications are also found at PIR-associated TSRs.

In their study mapping genome-wide RNA PolII peaks, in addition to dSSRs, Cordon-Obras et al. identified RNA PolII peaks at PIRs in *T. brucei*. The authors reported two such PIRs to demonstrate promoter activity in reporter assays (30). Our study, along with the studies in *T. brucei* by Wedel et al. and Cordon-Obras et al. (4, 30), also reinforces the fact that while no consensus sequences have been identified across dSSRs/HT sites, at least some transcriptional events in these parasites are governed by defined *cis*-acting elements. Wedel et al., who analyzed genome-wide maps of nucleosomal occupancy and RNA PolII enrichment positions in *T. brucei* and conducted further investigations using reporter assays, uncovered a GT-rich motif at the dSSRs that promoted directional transcription initiation in reporter assays (4). In focusing on TSRs lying in PIRs, the present study has also uncovered the presence of a GT-rich motif at these PIRs, identifying at least one shared feature between TSRs at dSSRs and PIRs. Using reporter assays, Cordon-Obras et al. determined that bidirectional transcription from dSSRs was due to two separate promoter regions, each promoter driving unidirectional transcription of one PTU. In dissecting one such unidirectional promoter by creating a panel of nested deletions, the core promoter was narrowed down to the 75 bp region immediately upstream of the first gene of the PTU. Three motifs critical for driving transcription were identified in this core promoter (30). A more recent study in *Trypanosoma cruzi* has uncovered distinct conserved motifs in the TSRs of PTUs, although the roles of these motifs in driving transcription have not been validated (35).

The findings from the present study in *Leishmania donovani,* along with the studies by Wedel et al. and Cordon-Obras et al., collectively counter the long-standing belief that transcription in trypanosomatids is only constitutive, and no *cis*-element-dependent regulation occurs at the step of transcription initiation. Our work underscores the fact that the regulation of gene expression in these organisms is a complex phenomenon, involving an unusual genome organization coupled to polycistronic transcription, as well as monocistronic transcription, with *cis*-acting elements regulating at least some transcriptional activation events, in addition to epigenetic regulation and regulation by gene dosage. While our findings suggest the parasite exhibits cell cycle-dependent monocistronic transcription of a small subset of genes, several interesting questions arise: how widespread is this second tier of transcription across the genome? What epigenetic marks exist at these TSRs, and how do they compare with those found at dSSRs? Transcription termination sites at cSSRs are marked and regulated by the presence of an unusual residue: Base J (36). What regulates transcription termination in the case of TSRs at PIRs, and how does polycistronic transcription move past these sites? What proteins bind to the sequences at the TSRs identified at PIRs? These and other questions that remain to be investigated further in in-depth studies underline the complexities of transcription and gene regulation in this family of unicellular parasites.

## Data Availability

The authors declare that all data are available within the paper and the Supplementary Material files.

## SUPPLEMENTAL MATERIAL

Supplemental figures and tables spectrum.01096-26-s0001.pdf Figures S1 to S6, and Tables S1 and S2.

Figure S7 spectrum.01096-26-s0002.xlsx Raw data of Figure 2.

Figure S8 spectrum.01096-26-s0003.xlsx Raw data of Figure 3A.

Figure S9 spectrum.01096-26-s0004.xlsx Raw data of Figure 5.

Figure S10 spectrum.01096-26-s0005.xlsx Raw data of Figure 7.

Figure S11 spectrum.01096-26-s0006.xlsx Raw data of Figure 8.

ASM does not own the copyrights to Supplemental Material that may be linked to, or accessed through, an article. The authors have granted ASM a non-exclusive, world-wide license to publish the Supplemental Material files. Please contact the corresponding author directly for reuse.
