# Supplementary material for: Transcription Start Regions in PTU-intergenic regions drive cell cycle-dependent transcriptional activation events in Leishmania donovani
Source: Microbiol Spectr. 2026 Aug 3;14(9):e01096-26. doi: 10.1128/spectrum.01096-26 (PMC13532274; doi:10.1128/spectrum.01096-26)
Supplement: Supplementary file 1 [file spectrum.01096-26-s0001.pdf]

Transcription Start Regions in PTU-intergenic regions drive cell cycle dependent transcriptional activation events in *Leishmania donovani*

Ashish Singh[1], Vishal Dashora[1], Arushi Khanna[2] and Swati Saha*

Department of Microbiology

University of Delhi South Campus

New Delhi-110021

INDIA

[2]Current affiliation:

New Delhi Tuberculosis Centre

Delhi-110002

INDIA.

[1]*These two authors contributed equally to the work*

To whom correspondence may be addressed

Running title: *Leishmania* transcription initiation from sites within PIRs

Keywords: *Leishmania donovani*, trypanosomatid, *Leishmania* transcription, *cis*-acting regulation, *Leishmania* gene regulation

Figure S1

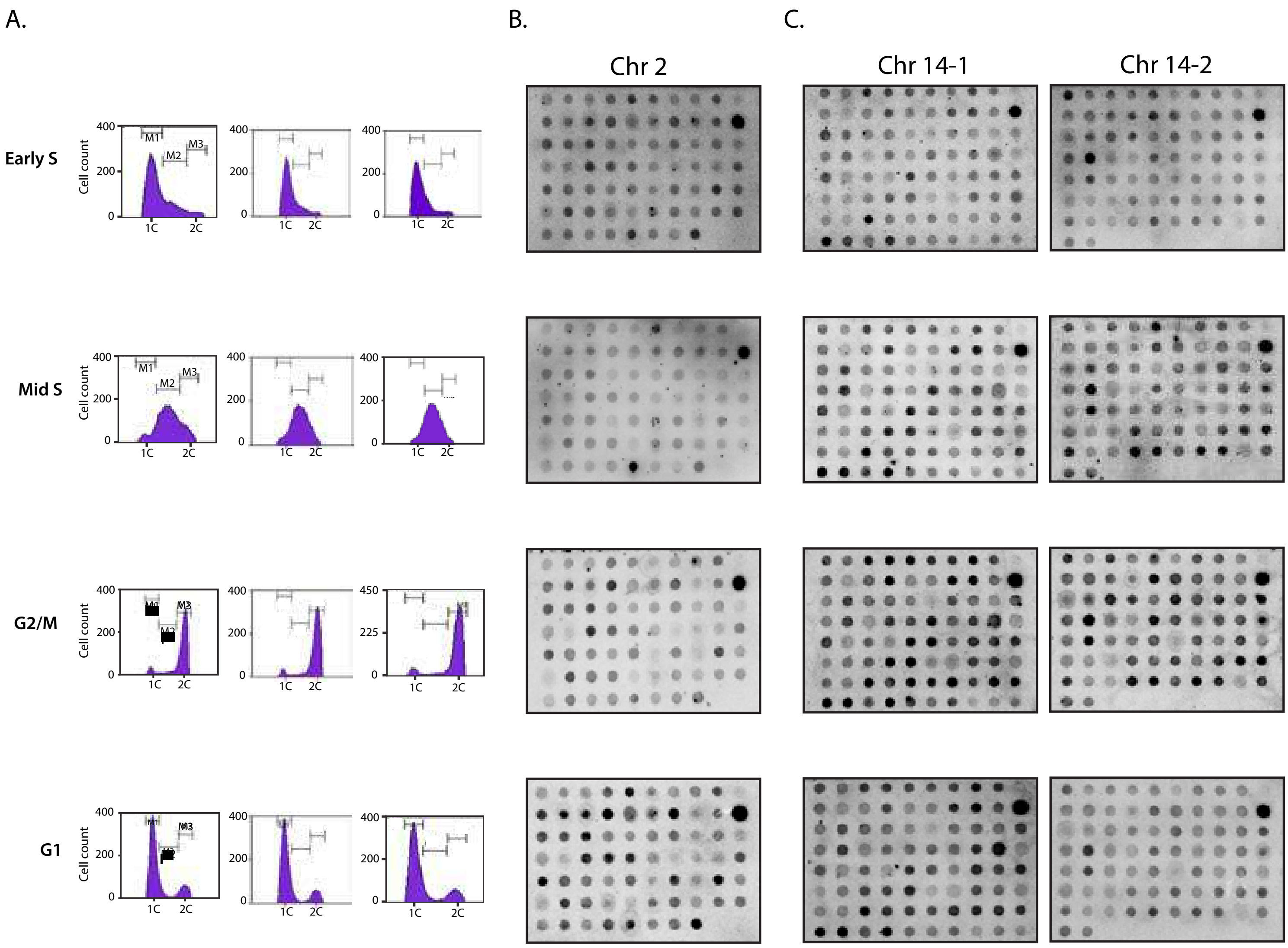

Figure S1: A. Run-on assays using synchronized *Leishmania* parasites at different cell cycle stages. Radiolabelled nascent transcripts were analyzed using dot blot hybridizations, followed by phosphorimaging. *A.* Histograms indicate the flow cytometry profiles of the parasites at the time of performance of the run-on reactions. Three reactions were carried out at each stage, and nascents pooled before hybridization. Stage at which nascents were isolated are indicated on the left-hand side of each row. *B.* Column of blots representing analyses of nascent transcripts of chromosome 2 at the different cell cycle stages, as indicated in the particular rows in *A*. *C.* Columns of blots representing analyses of nascent transcripts of chromosome 14 at the different cell cycle stages, as indicated in the particular rows in *A*.

Figure S2

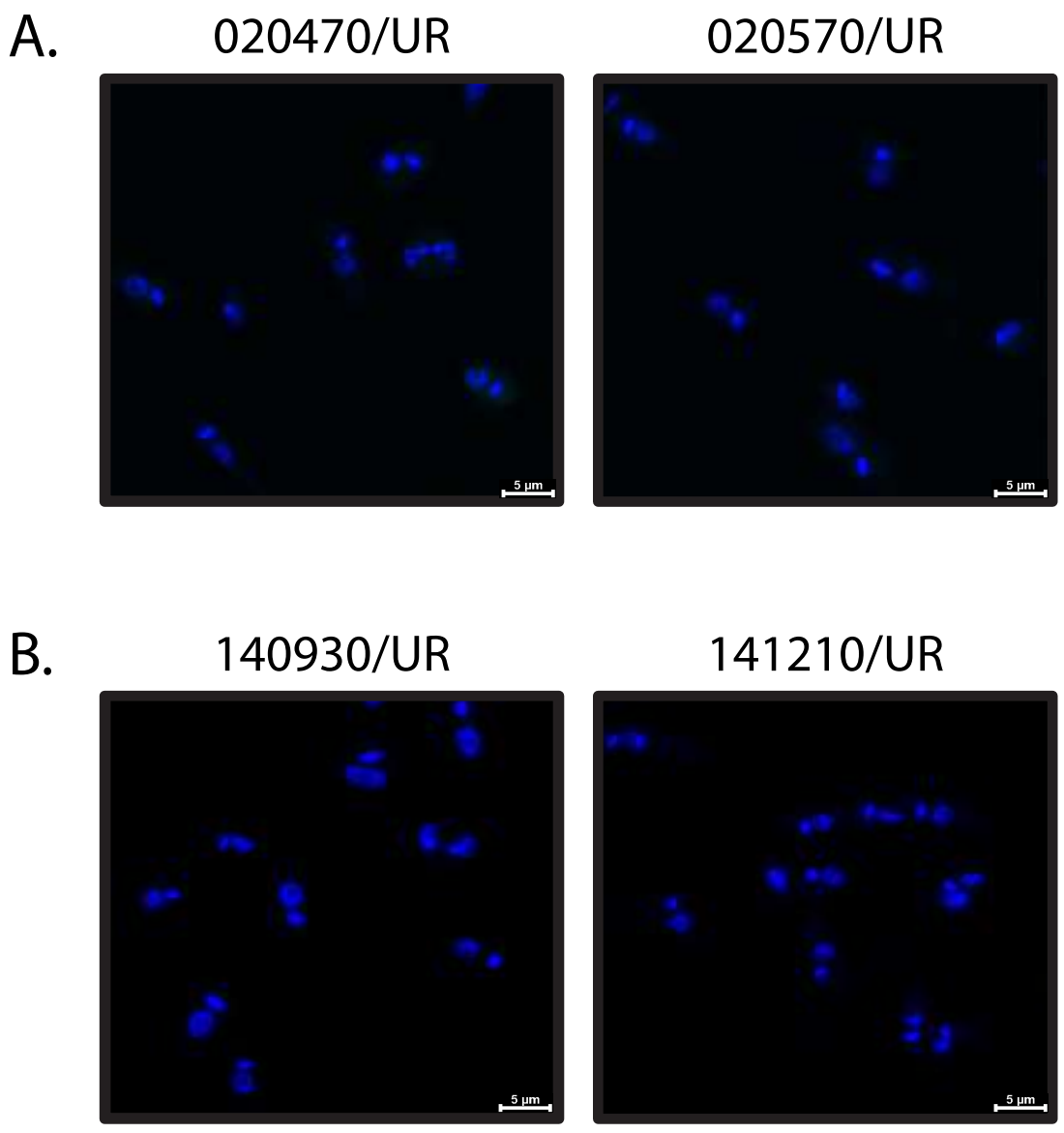

Figure S2: Microscopic analysis of reporter expression. A. using URs of chromosome 2 genes. B. using URs of chromosome 14 genes. No eGFP expression was detected in these cases.

Figure S3

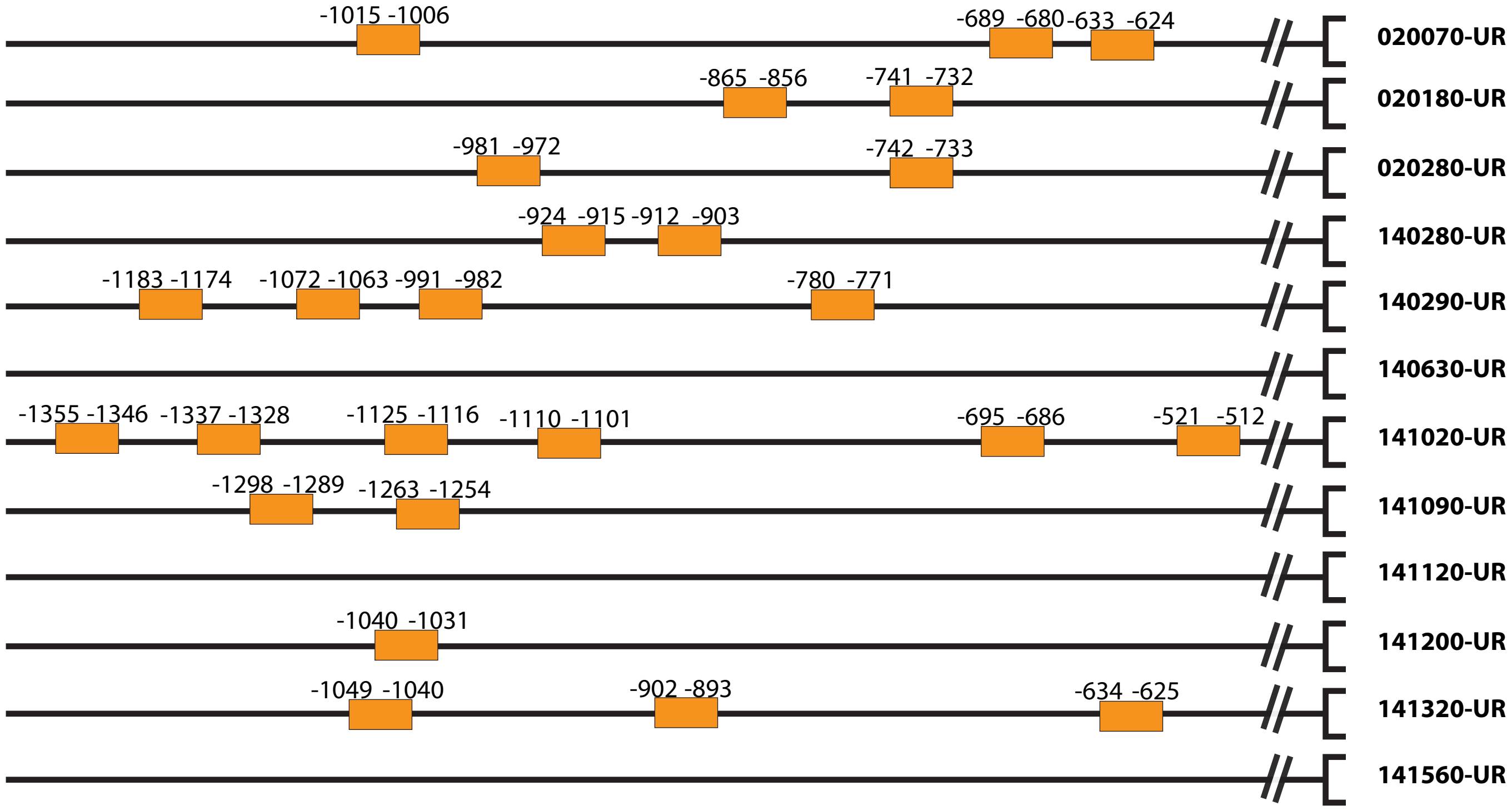

Figure S3: Analysis of the 12 TSRs. Regions [-500 to -1500] depicted here by black line, with labels on the side indicating the gene number whose TSR is being analyzed. Motif I: 10-mer GT-rich sequence, indicated by orange boxes on black line. Positions of motifs indicated by numerics above boxes, where -1 refers to the first nucleotide upstream of the gene start codon.

Figure S4

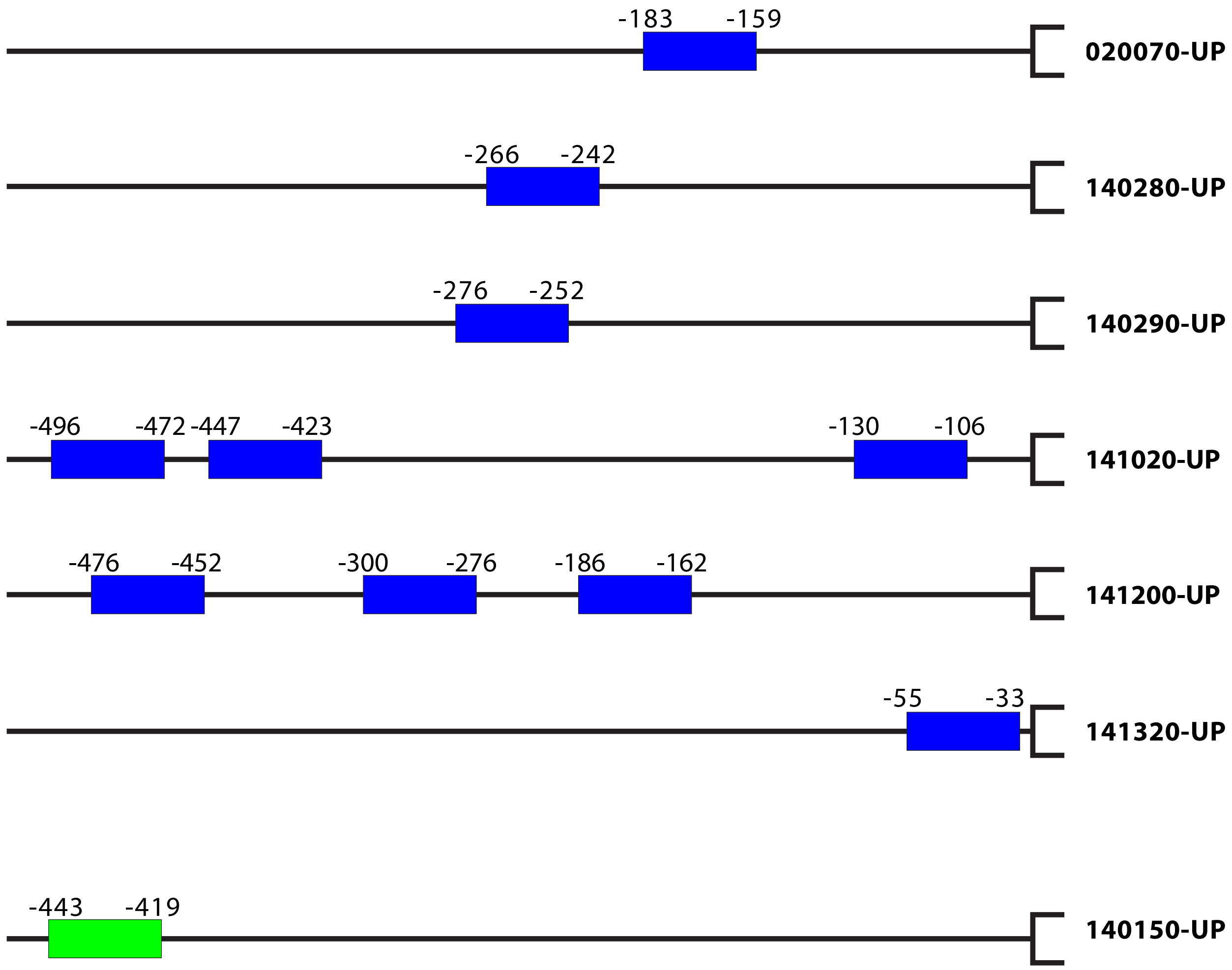

Figure S4: Identification of Motif II in the TSRs and non-activated URs. Regions [-1 to -500] depicted here by black line, with labels on the side indicating the gene number whose TSR is being analyzed. Motif II indicated by blue boxes on black line in case of the TSRs, and by green box on black line in case of non-activated UR. Non-activated URs analyzed: 020080, 020420, 020530, 140020, 140110, 140150, 140820, 140840, 140860, 141060, 141270, 141390. Positions of motifs indicated by numerics above boxes, where -1 refers to the first nucleotide upstream of the gene start codon.

Figure S5

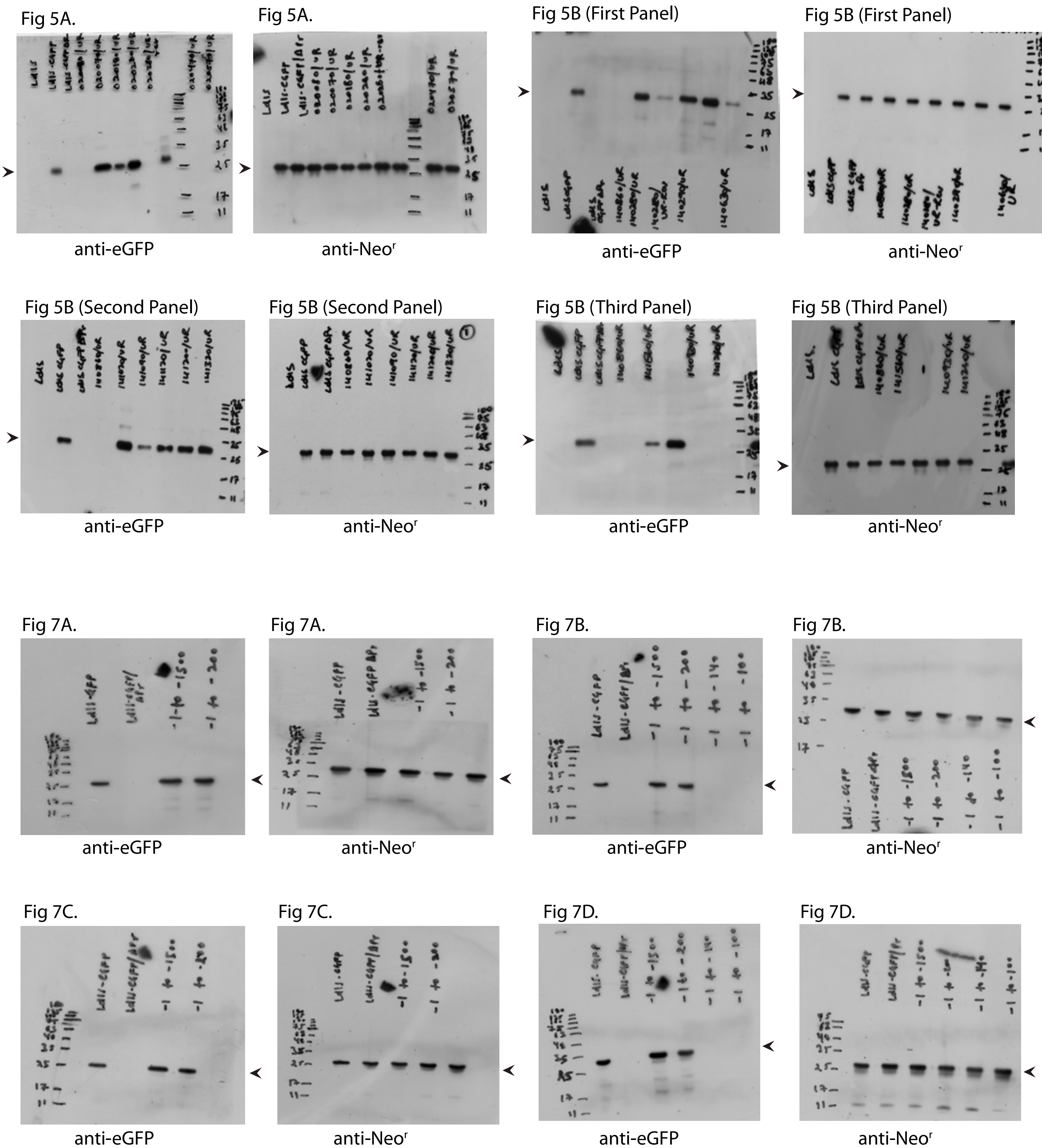

Figure S5: Uncropped blot images of Figures 5 and 7. Each blot is labelled with the figure number whose uncropped blot it shows.

Figure S6

Fig 8A.

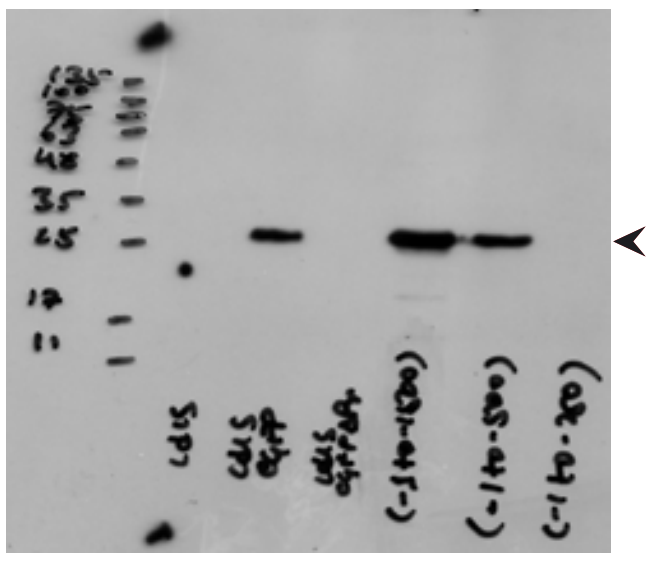

anti-eGFP

Fig 8A.

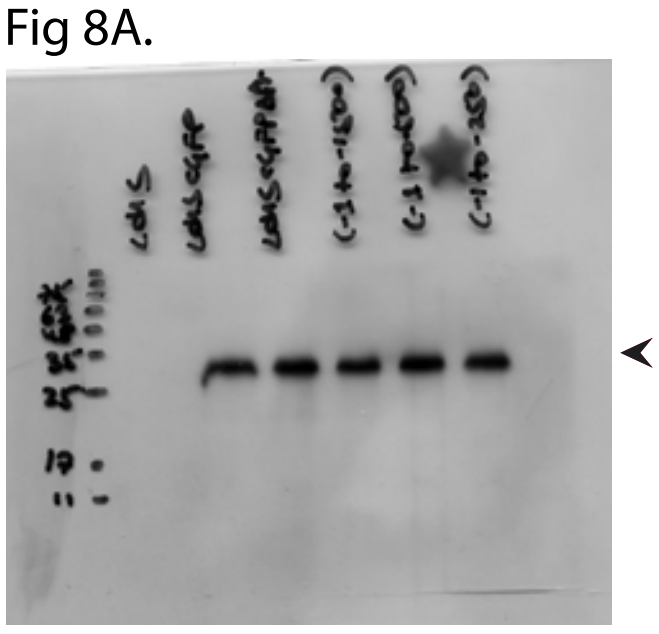

anti-Neo^r

Fig 8B.

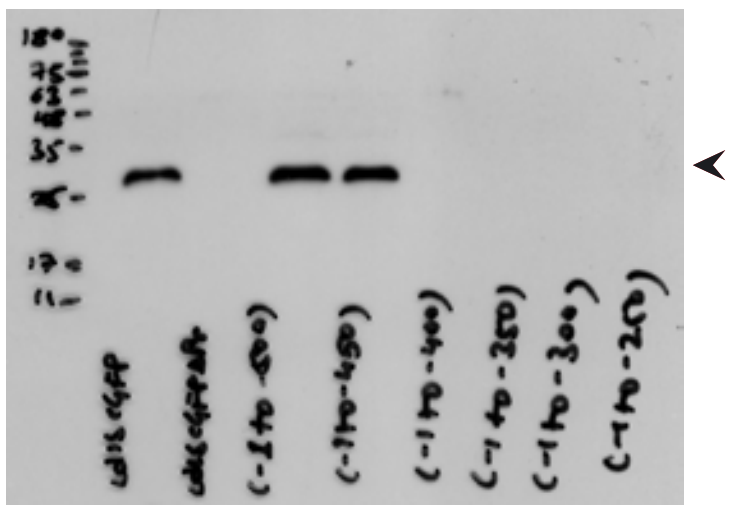

anti-eGFP

Fig 8B.

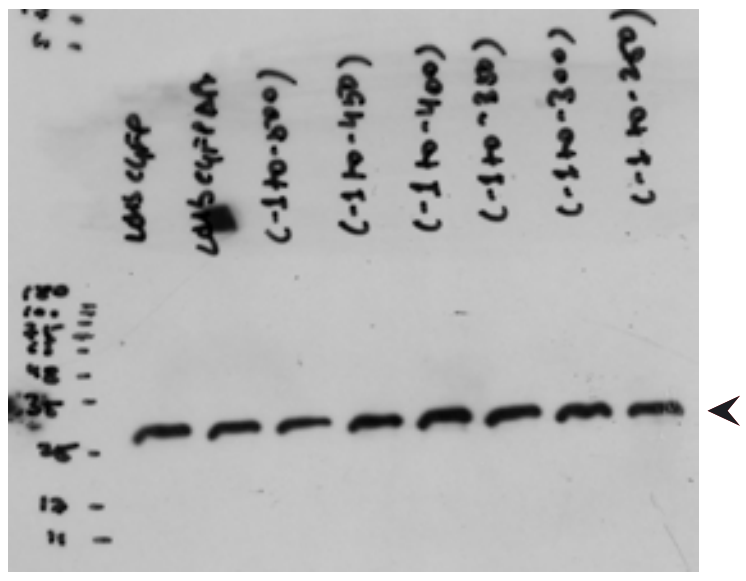

anti-Neo^r

Fig 8C.

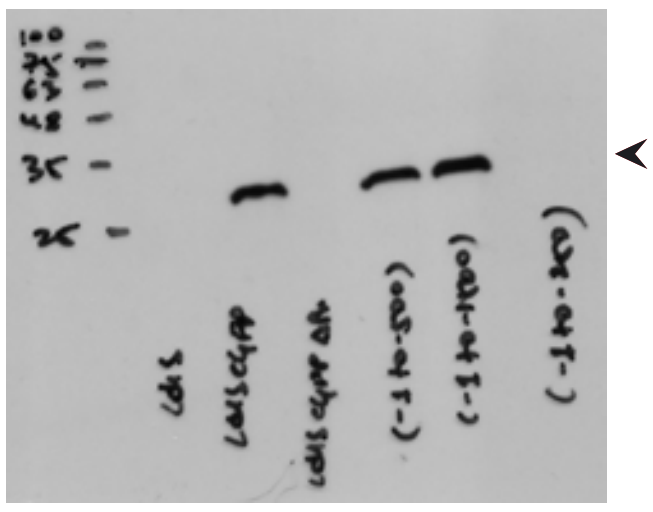

anti-eGFP

Fig 8C.

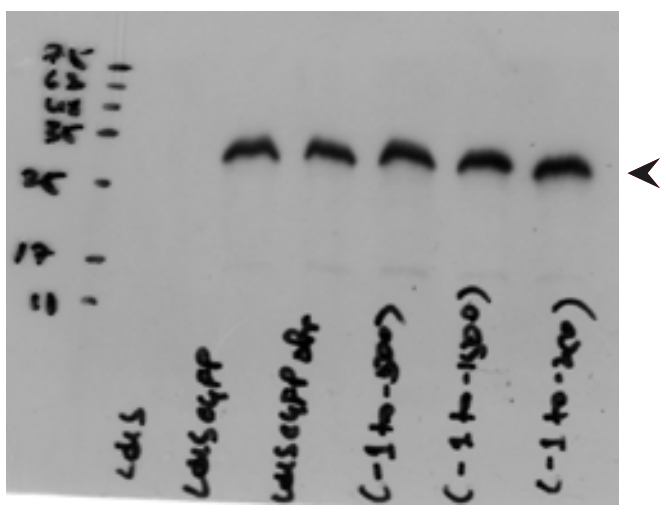

anti-Neo^r

Fig 8D.

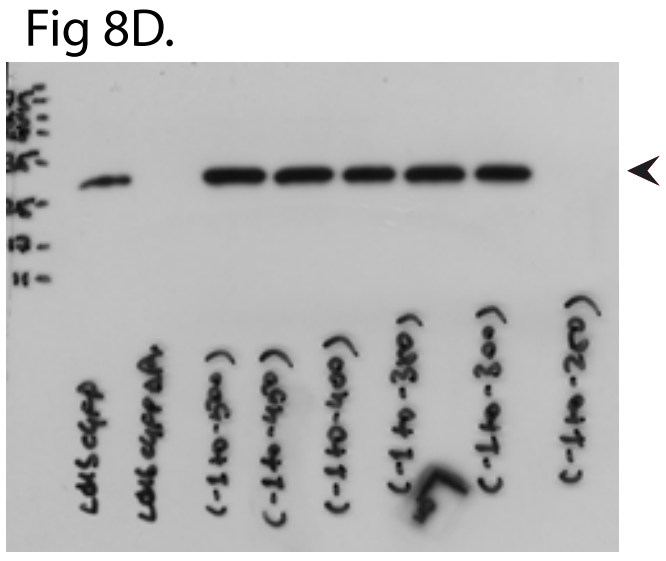

anti-eGFP

Fig 8D.

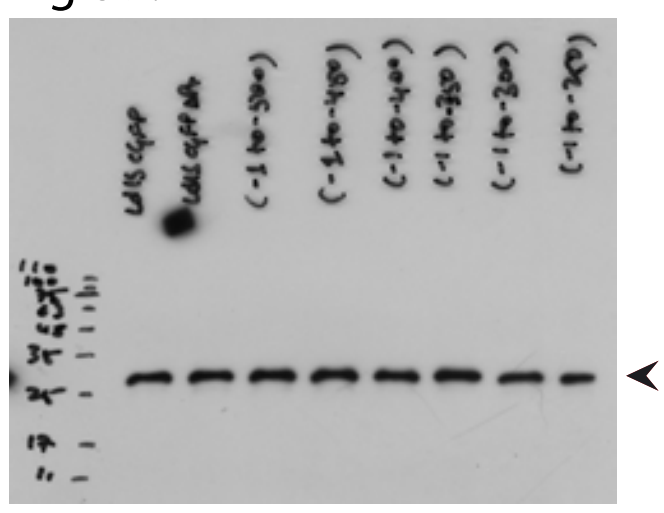

anti-Neo^r

Fig 9A.

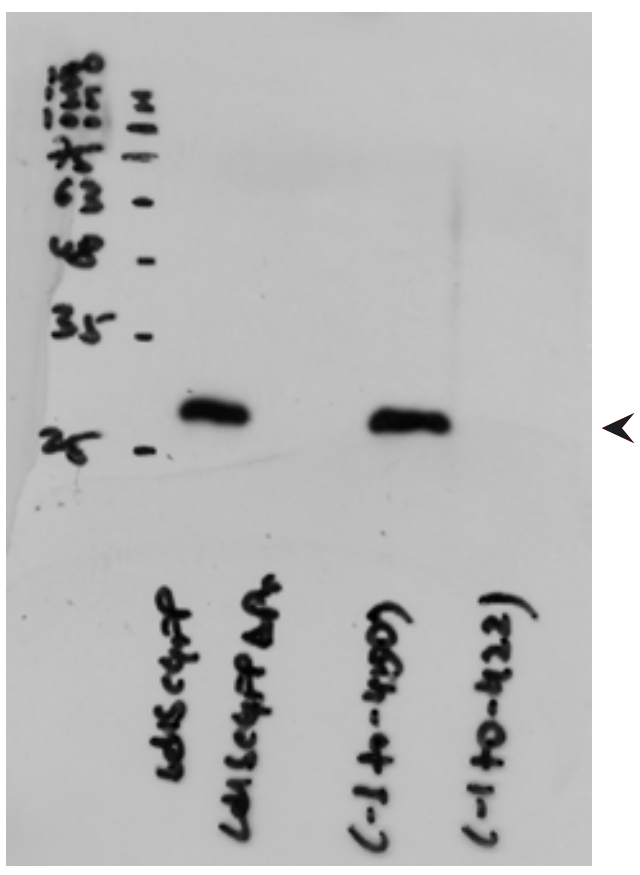

anti-eGFP

Fig 9A.

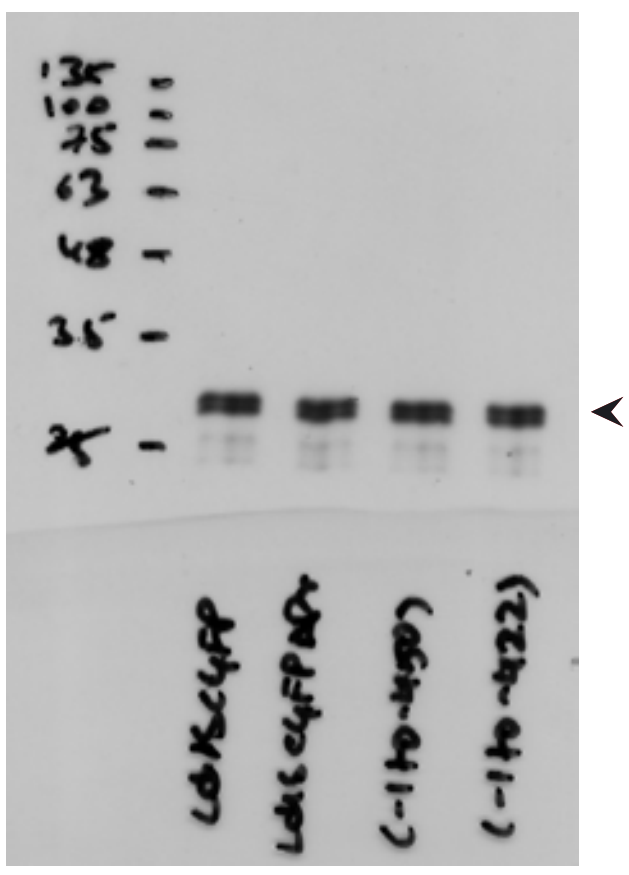

anti-Neo^r

Fig 9B.

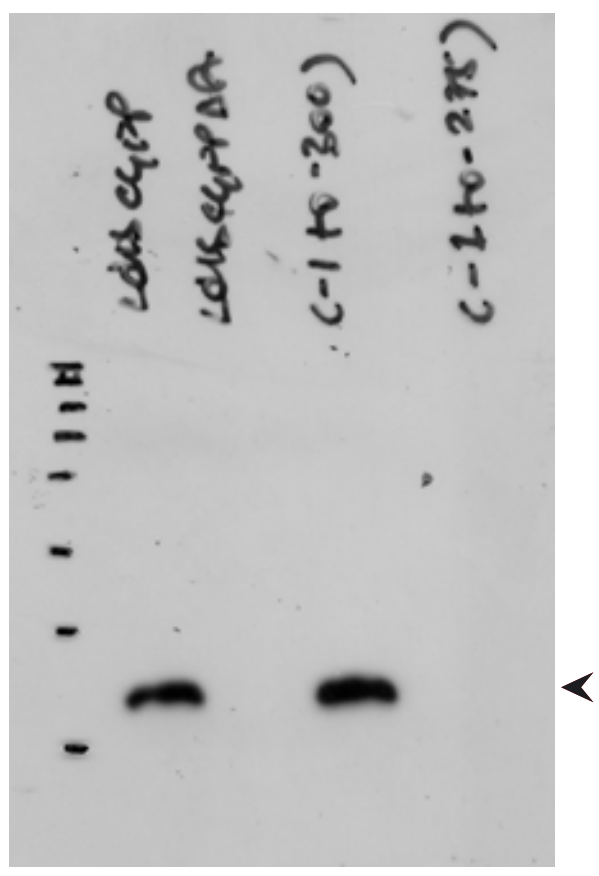

anti-eGFP

Fig 9B.

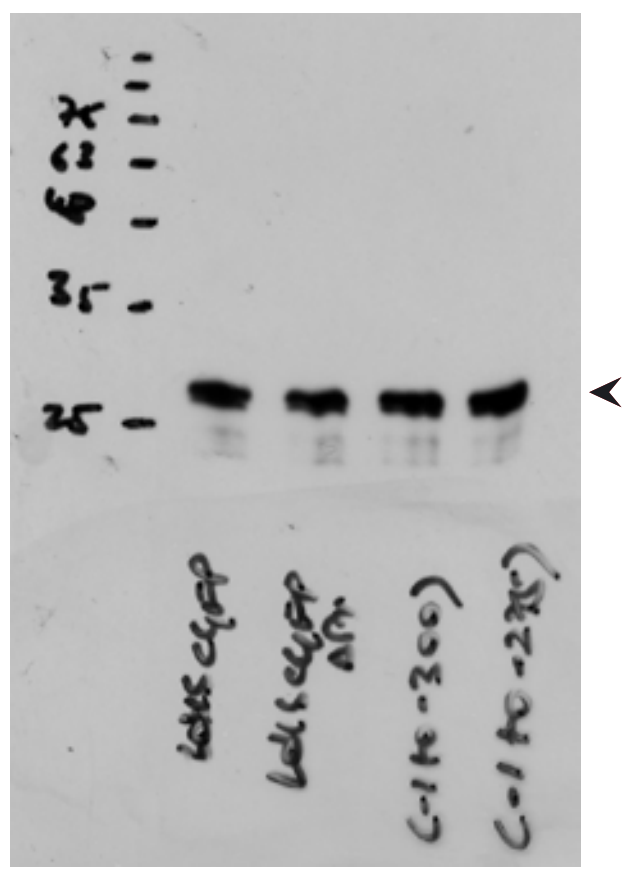

anti-Neo^r

Figure S6: Uncropped blot images of Figures 8 and 9. Each blot is labelled with the figure number whose uncropped blot it shows.

Table S1: List of Motif I sequences in the 12 TSRs

S.No	UR	Strand	Position		Sequence
			Start	End	
1	020070	+	-1015	-1006	TGTGTGTGTG
2	020070	+	-152	-143	TGTGTGCGTG
3	020070	+	-633	-624	TGTGTGCGCG
4	020070	+	-344	-335	TGCGTGTATG
5	020070	+	-689	-680	TGTGCGTCTG
6	020180	+	-14	-5	TGTGTGTGTG
7	020180	+	-389	-380	TGTGTGTGTG
8	020180	+	-741	-732	TGCATGCGTG
9	020180	+	-88	-79	TGCGCGCGCG
10	020180	+	-865	-856	TGTGTGCCCG
11	020280	+	-981	-972	TGCGTGCGTG
12	020280	+	-742	-733	GGTGTGCGTG
13	140280	+	-912	-903	TGTGTGTGTG
14	140280	+	-924	-915	TGTGTGTGTG
15	140290	+	-430	-421	TGTGTGTGTG
16	140290	+	-780	-771	TGTGTGCGTG
17	140290	+	-991	-982	TGTGTGCGCG
18	140290	+	-1183	-1174	TGTGTGCTTG
19	140290	+	-1072	-1063	TGTGCGCGCG
20	140290	+	-40	-31	TGTGCGCATG
21	140630	+	-295	-286	TGCGTGTTTG
22	141020	+	-1337	-1328	TGTGTGTGTG
23	141020	+	-1355	-1346	TGTGTGTGTG
24	141020	+	-695	-686	TGTGCGTGTG
25	141020	+	-1110	-1101	TGCGTGCGTG
26	141020	+	-1125	-1116	TGCGTGTATG
27	141020	+	-521	-512	TGCGTGTCTG
28	141090	+	-151	-142	TGTGTGCGCG
29	141090	+	-1263	-1254	TGTGTGCGCG
30	141090	+	-303	-294	TGTGCGTGCG
31	141090	+	-1298	-1289	TGCGCGTATG
32	141200	+	-341	-332	TGCGTGCGTG
33	141200	+	-456	-447	TGTGTGGGTG
34	141200	+	-1040	-1031	TGCGTGCGCG
35	141320	+	-634	-625	TGTGTGTGTG
36	141320	+	-1049	-1040	TGTGTGTGTG
37	141320	+	-487	-478	TGTGCGCGCG
38	141320	+	-902	-893	TGTGCGCGCG
39	141560	+	-371	-362	TGTGTGCATG

Table S1: Table indicates the positions of the Motif I sequences found in the [-1 to -1500] regions of the 12 TSRs.

Table S2: List of Motif II sequences in the TSRs / non-activated UR.

S. No.	Upstream regions	Strand	Position		p-value
			Start	End	
**TSRs**					
1	020070	+	-183	-159	3.10E-07
2	140280	+	-266	-242	8.23E-07
3	140290	+	-276	-252	4.38E-07
4	141020	+	-496	-472	4.61E-10
5	141020	+	-447	-423	1.14E-08
6	141020	+	-131	-107	4.81E-07
7	141200	+	-476	-452	1.34E-09
8	141200	+	-300	-276	1.06E-07
9	141200	+	-186	-162	3.32E-07
10	141320	+	-57	-33	8.09E-07
**UR of non-activated gene**					
11	140150	+	-443	-419	6.74E-08

Table S2: Table indicates the positions of the Motif II sequences found in the [-1 to -500] regions of the TSRs/ non-activated URs.
